# Extracellular peptide Kratos restricts cell death during vascular development and stress in Arabidopsis

**DOI:** 10.1093/jxb/erz021

**Published:** 2019-02-07

**Authors:** Sacha Escamez, Simon Stael, Julia P Vainonen, Patrick Willems, Huiting Jin, Sachie Kimura, Frank Van Breusegem, Kris Gevaert, Michael Wrzaczek, Hannele Tuominen

**Affiliations:** 1 Umeå Plant Science Centre, Department of Plant Physiology, Umeå University, Umeå, Sweden; 2 Ghent University, Department of Plant Biotechnology and Bioinformatics, Technologiepark, Ghent, Belgium; 3 VIB-UGent Center for Plant Systems Biology, Technologiepark, Ghent, Belgium; 4 Department of Biochemistry, Ghent University, Ghent, Belgium; 5 VIB-UGent Center for Medical Biotechnology, Ghent, Belgium; 6 Organismal and Evolutionary Biology Research Programme, Viikki Plant Science Centre, VIPS, Faculty of Biological and Environmental Sciences, University of Helsinki, Helsinki, Finland

**Keywords:** Arabidopsis, autophagy, cell death, peptide, peptidomics, programmed cell death, stress response, vascular development, xylem

## Abstract

During plant vascular development, xylem tracheary elements (TEs) form water-conducting, empty pipes by genetically regulated cell death. Cell death is prevented from spreading to non-TEs by unidentified intercellular mechanisms, downstream of METACASPASE9 (MC9)-mediated regulation of autophagy in TEs. Here, we identified differentially abundant extracellular peptides in vascular-differentiating wild-type and *MC9*-down-regulated Arabidopsis cell suspensions. A peptide named Kratos rescued the abnormally high ectopic non-TE death resulting from either *MC9* knockout or TE-specific overexpression of the ATG5 autophagy protein during experimentally induced vascular differentiation in Arabidopsis cotyledons. Kratos also reduced cell death following mechanical damage and extracellular ROS production in Arabidopsis leaves. Stress-induced but not vascular non-TE cell death was enhanced by another identified peptide, named Bia. Bia is therefore reminiscent of several known plant cell death-inducing peptides acting as damage-associated molecular patterns. In contrast, Kratos plays a novel extracellular cell survival role in the context of development and during stress response.

## Teaser

During development or stress response, plants need mechanisms to restrict cell death to the target cells. Kratos is the first plant extracellular peptide found to restrict cell death.

## Introduction

Genetically regulated cell death has important roles in stress responses, immunity, and development of multicellular organisms (Clarke and Clarke, 1996; Inohara and Nuñez, 2003; Galluzzi *et al.*, 2015; Daneva *et al.*, 2016; Huysmans *et al.*, 2017). Upon stress, infection, or developmental stimuli, specific cells become committed to cell death and organized dismantlement. Such cell elimination events, unlike purely accidental cell death, rely on genetic regulation and occur in an anatomically organized manner often referred to as programmed cell death (PCD) (Lockshin and Williams, 1964; Lockshin and Zakeri, 2001; van Doorn *et al.*, 2011; Galluzzi *et al.*, 2015). In contrast to PCD, which implies a mechanism of cell suicide (Reynolds, 2014), cell death can also spread from target cells to neighbouring cells through non-cell autonomous mechanisms (Galluzzi *et al.*, 2015; Escamez and Tuominen, 2017). In animals, a well-known example of such spreading cell death occurs during inflammation (Wallach *et al.*, 2014; Kang *et al.*, 2015; Zhong *et al.*, 2016; Deretic and Levine, 2018). Generally, this latter type of ‘runaway’ cell death, here referred to as ‘ectopic’ cell death, is also genetically regulated even though the underlying molecular mechanisms in plants are poorly understood (Jabs *et al.*, 1996; Endo *et al.*, 2001; Mach *et al.*, 2001; Brodersen *et al.*, 2002; Tuominen *et al.*, 2004; Overmyer *et al.*, 2005; Wrzaczek *et al.*, 2009, 2015; Coll *et al.*, 2010; Escamez *et al.*, 2016).

While inflammation in animals relies on the same core machinery in different situations (Wallach *et al.*, 2014; Kang *et al.*, 2015; Zhong *et al.*, 2016; Deretic and Levine, 2018), the known instances of plant ectopic cell death seem to involve very different extracellular factors depending on the context (Endo *et al.*, 2001; Tuominen *et al.*, 2004; Overmyer *et al.*, 2005; Wrzaczek *et al.*, 2009, 2015; Escamez *et al.*, 2016). During the formation of the plant vascular xylem tissue, the water-conducting tracheary element (TE) cells rid themselves of their protoplasts to form pipe-like structures by undergoing developmental cell death (Escamez and Tuominen, 2014). Dying TEs release molecules that can be harmful to the neighbouring non-TE cells, and restricting subsequent ectopic non-TE cell death requires active regulation (Endo *et al.*, 2001; Escamez *et al.*, 2016). We had previously found that ectopic non-TE cell death increased upon RNAi-based down-regulation of the TE-expressed Arabidopsis caspase structural homolog METACASPASE9 (MC9) in xylem-differentiating cell suspensions (Escamez *et al.*, 2016).

*MC9* down-regulation was also associated with an apparent increase in autophagy specifically in the TE cells (Escamez *et al.*, 2016). Autophagy is an evolutionarily conserved cellular process that targets cellular components for degradation, either in bulk or in a targeted manner, as a means of post-translational regulation or for recycling purposes (Bozhkov, 2018; Marshall and Vierstra, 2018). Strikingly, TE-specific down-regulation of the autophagy protein ATG2 in the *MC9*-down-regulated cells was found to reduce ectopic non-TE death back to wild-type levels (Escamez *et al.*, 2016). It therefore seems that ectopic non-TE cell death is regulated by the level of autophagy in TEs, downstream of MC9 (Escamez *et al.*, 2016). The mechanism behind this apparent regulation of autophagy by MC9 remains unknown. None of the core autophagy machinery proteins (Marshall and Vierstra, 2018) appears among the identified targets from MC9-mediated proteolysis, and only a single isoform of one autophagy protein (ATG18b) contains putative MC9 target sites (Tsiatsiani *et al.*, 2013). Arabidopsis MC9 and its orthologues in other species have glyceraldehyde-3-phosphate dehydrogenase (GAPDH) as an evolutionarily conserved target (Tsiatsiani *et al.*, 2013). GAPDH has been implicated in the regulation of autophagy in several species (Colell *et al.*, 2007; Tristan *et al.*, 2011; Henry *et al.*, 2015), but whether GAPDH could represent the missing link between MC9 and autophagy, upstream of ectopic cell death, has not been investigated.

Ectopic non-TE death in xylem-differentiating cell suspensions is regulated by MC9 and autophagy in TEs, which implies that ectopic non-TE cell death is regulated in a non-cell-autonomous manner. We therefore sought to identify downstream, extracellular factors that mediate the MC9- and autophagy-dependent regulation of ectopic non-TE death. With MC9 being a protease, and autophagy being involved in secretion of proteins and peptides (e.g. during inflammation in animals) (Kimura *et al.*, 2017*a*,b; Zhang *et al.*, 2017), we hypothesized that extracellular peptides could function to regulate ectopic non-TE death downstream of MC9 and autophagy.

## Materials and methods

### Plant material and growth conditions

Cell suspensions from the Col-0 wild-type background were previously described (Escamez *et al.*, 2016; Pesquet *et al.*, 2010). *MC9*-RNAi cell suspensions were generated as previously described (Escamez *et al.*, 2016) by *Agrobacterium*-mediated transformation of wild-type cell suspensions. Growth conditions and induction of vascular xylem-like differentiation were as previously described (Escamez *et al.*, 2016).

Arabidopsis genotypes were all in the Col-0 background. The T-DNA knockout line *mc9-2* (SALK_075814) has been previously published (Bollhöner *et al.*, 2013). The T-DNA lines *kratos-1* (SALK_201112) and *bia-1* (SALK_069212) were obtained from the Nottingham Arabidopsis Stock Centre (NASC), and homozygous plants were identified that did not display expression of the corresponding genes (Supplementary Fig. S1 at *JXB* online). For identification of knock-out mutants *kratos-1* and *bia-1*, the RNA was isolated with RNeasy plant kit (Qiagen), cDNA synthesis was performed with QuantiTect reverse transcription kit (Qiagen), and semi-quantitative PCRs were run with Go Taq Green Master Mix (Promega) according to manufacturer’s instructions (*Bia* forward primer: ATGACTCGAGGAAGTCAAAG; *Bia* reverse primer: TCACTTATTGTTTCCTTTGCCT; *Kratos* forward primer: ATGGGGCGTCTCGTTAGTG; *Kratos* reverse primer: TTAGTGGTGTCCGATTCCG). The transcriptional reporter lines pro*MC9::GUS* and pro*IRX1::GFP:GUS* were previously described (Bollhöner *et al.*, 2013; Escamez *et al.*, 2016). The vascularly expressed autophagasomal fluorescent marker *proATG8a::GFP:ATG8a* and the fluorescently labelled MC9 under the transcriptional control of the *MC9* endogenous promoter pro*MC9::MC9:mCherry* have been previously described (Bollhöner *et al.*, 2013; Furuta *et al.*, 2014).

The overexpressor of the autophagy rate-limiting ATG5 protein (Minina *et al.*, 2018) in TEs pro*IRX1::ATG5* was generated as follows. The *ATG5* full coding sequence was amplified by PCR, using primers including sequences for Gateway BP recombination (forward: GGGGACAAGTTTGTACAAAAAAGCAGCAGGCTTAATGGCG AAGGAAGCGGTCAAG; reverse: GGGGACCACTTTGTACAAGA AAGCTGGGTATCACCTTTGAGGAGCTTTCAC), and recombined using BP clonase into pDONR207 to generate a Gateway-compatible pENTR vector. The *IRX1* promoter fragment (pro*IRX1*; 1586 bp upstream of IRX1/AT4G18780) had previously been inserted instead of the 35S promoter fragment in the Gateway-compatible destination vector pK2GW7 (Karimi *et al.*, 2002; Escamez *et al.*, 2016). This pro*IRX1*-containing destination vector was recombined with the *ATG5*-containing pENTR vector using LR clonase, resulting in the pro*IRX1::ATG5* binary vector. *Agrobacterium tumefaciens* (stain GV3101) cells were electroporated with the pro*IRX1::ATG5* binary vector, and pro*IRX1::ATG5* Arabidopsis plants were generated by *Agrobacterium*-mediated transformation. pro*IRX1::ATG5* pro*ATG8a::GFP:ATG8a* was generated by crossing.

For induction of vascular differentiation in cotyledons using the Vascular Cell Induction Culture System Using Arabidopsis Leaves (VISUAL) system, both growth and induction conditions were extensively described previously (Kondo *et al.*, 2016). Each induction of one genotype in one treatment condition included four biological replicates (four seedlings in one and the same well). Plants used for stress-induced cell death assays were grown on soil (peat: vermiculite=1:1) under white luminescent light (220–250 µmol m^−2^ s^−1^) with a 12 h photoperiod (temperature 23/18 °C, relative humidity 70/90%) for 5 weeks.

### Isolation of extracellular peptides

Wild-type and *MC9*-RNAi cell suspensions were induced to differentiate (or not, as control) in triplicates, each replicate being in a volume of 200 ml (in 1 litre Erlenmeyer flasks). After 5 d, when the rate of TE differentiation is peaking (Escamez *et al.*, 2016), concomitantly with the rise of ectopic non-TE death in *MC9*-RNAi (Escamez *et al.*, 2016), the samples were harvested. The cells and extracellular medium of each sample were separated using inert sterile membranes with a 0.45 µm pore size (Millipore Stericup-HV Filter Units). The extracellular medium fractions were further filtered using Jumbosep centrifugation devices with a 3 kDa cut-off membrane (Pall) to separate the peptides (<3 kDa) from higher molecular mass (>3 kDa) protein (fragments). The higher molecular mass extracellular fraction was compared with cell lysates to verify the absence of detectable intracellular contaminations in the extracellular medium (Supplementary Fig. S2A). Peptides in the low molecular mass (<3 kDa) extracellular fraction were further purified by reverse-phase C18 solid phase extraction (SepPak C18, Waters) and the resulting samples were freeze-dried.

### Peptide identification and relative quantification

Freeze-dried peptide samples were dissolved in 10 µl of 50% acetonitrile (ACN) and subsequently diluted in 200 µl ultrapure water and acidified with trifluoroacetic acid (TFA) to a 1% (v/v) final concentration. No digestion by trypsin or other enzymes was performed, this to preserve the structure of the endogenously generated peptides. Ten microlitres of each sample was subjected to liquid chromatography–tandem mass spectrometry (LC-MS/MS) analysis using a Q Exactive mass spectrometer (Thermo Fisher Scientific) that was operated as previously described (Stes *et al.*, 2014).

Raw mass spectrometry data were searched against TAIR10 representative proteins and contaminant protein sequences by the built-in Andromeda search engine of the MaxQuant software (v1.5.3.30) (Cox and Mann, 2008). The ‘unspecific cleavage’ setting was selected in MaxQuant as no protease was used to prepare the samples. The minimum peptide length was set to eight amino acids, matching-between-runs was enabled with an alignment time of 30 min and matching time window of 30 s. Variable modifications were oxidation of methionine and proline (+15.995 Da) and protein N-terminal acetylation (+42.011 Da). The identified peptides (false discovery rate <0.01) and their respective intensities were extracted from the ‘peptides.txt’ output file. As trimming of N- and C-terminal ends of peptides was frequently observed, we grouped overlapping peptides to a single longest peptide variant (LPV) (Secher *et al.*, 2016). More specifically, if a peptide sequence was contained within another peptide or extended another peptide by up to three amino acids, both peptides were grouped to a single LPV. The intensity of a LPV is the sum of the intensities of its constitutent peptides.

In the next phase, the Perseus (v1.5.4.0) computational platform (Tyanova *et al.*, 2016) was used for quantitative analysis of the peptidomics data. Peptides matching contaminants, decoy proteins, or without an intensity value were omitted, resulting in a list of 689 peptides (Supplementary Dataset S1). For statistical analyses, only peptides with at least intensity values for two biological replicates in at least one condition were included, resulting in 201 unique peptides compared with statistical tests. Peptide intensities were log10 transformed and missing values were imputed for the total matrix from the normal distribution with a downshift of 1.8 and width of 0.3. A two sample Student’s *t*-test was performed for pairwise comparisons and the interaction between the condition (induced or not) and genotype effects was tested by a two-way ANOVA.

The mass spectrometry proteomics data have been deposited at the ProteomeXchange Consortium via the PRIDE (Vizcaíno *et al.*, 2016) partner repository with the dataset identifier PXD010886.

### Preparation of peptide solutions

Crude, unpurified (35%<purity<70%, depending on the peptide) peptide synthesis solutions for all the candidate peptides (Supplementary Table S1) as well as high purity peptide solutions (>95%) for peptides 3, 4, Kratos and Bia were obtained from GeneCust. All the peptides were dissolved in 10 mM phosphate buffer pH 7 to 1 mM master stocks, taking into account peptide purity in the crude extract to actually obtain 1 mM peptide solutions. Whenever a peptide was visibly insoluble at pH 7, the pH of the master solutions was either lowered with formic acid or increased with ammonia until dissolution was observed. From the 1 mM peptide solutions, master aliquots of 1, 10 and 50 µM (1000× solutions) were prepared for experimental use without repeating freezing–thawing cycles.

### Microscopy and image analyses

Samples for promoter–reporter assays with β-glucuronidase (GUS) staining were prepared as previously described (Bollhöner *et al.*, 2013). All other cotyledon samples (VISUAL) were fixed and cleared prior to microscopy observation as previously described (Kondo *et al.*, 2016).

All micrographs used for display and quantifications were acquired using an Axioplan II epifluorescence upright microscope equipped with an Axiocam HR color camera. The same imaging settings were conserved for all fluorescence micrographs used for quantifications of TE differentiation and ectopic non-TE death. For instance, images were acquired with a ×4 lens, a SOLA SM II LED light source was used for excitation, a dichroic beam splitter allowed blue light excitation (450–490 nm) and long pass detection of all emission fluorescence from green to red (long pass from 515 nm onward), and an exposure time of 380 ms was used.

Quantification of cotyledon areas covered with GUS staining, with TEs, or with dead non-TEs was performed by manually selecting and measuring the corresponding regions of interest (ROIs) in the open source software ImageJ. Especially, identification of TE and dead non-TE autofluorescence required manual input from the experimenters due to the fact that the numerous variable features of autofluorescence (cell size, cell shape, layering of cells, autofluorescence intensity, patterned secondary walls, etc.) did not allow for fully automatized segmentation pipelines (Supplementary Fig. S3N, O). As a result, autofluorescent dead non-TEs were manually identified and selected using the ‘selection brush tool’ in ImageJ, by several experimenters in parallel and with high number of experiment replication to ensure reproducibility. The selected dead non-TE autofluorescence area was quantified with ImageJ, as well as the total autofluorescence area of each cotyledon, allowing us to deduce the TE autofluorescence area by subtracting the dead non-TE area from the total autofluorescence area.

Confocal laser scanning microscopy analyses were performed as described previously (Escamez *et al.*, 2016), using an inverted Zeiss LSM780 confocal microscope. Imaging for colocalization purposes was performed with a ×40 water immersion lens by simultaneous excitation (due to the rapid movement of autophagosomes) of green fluorescent protein (GFP) and mCherry with 488 nm and 561 nm lasers, respectively, with a MBS 488/561 beam splitter, and emission detection windows of 495–546 nm and 595–656 nm, respectively. Colocalization analyses were performed using ImageJ with the Pearson–Spearman Colocalization (PCS) plugin. For counting of GFP:ATG8a puncta, all compared images within an experiment were acquired with the same settings. To distinguish the puncta from the diffuse GFP fluorescence background, a single threshold for fluorescence intensity in the GFP channel was applied to all the images from a single experiment.

### Stress-induced cell death assays

Mechanical induction of cell death was performed similarly to a previously described study (Wrzaczek *et al.*, 2009). Briefly, fully expanded leaves of 5-week-old Col-0 or *mc9-2* plants were infiltrated with 50 nM Kratos, Bia, or phosphate buffer as a control. Leaf disks were cut from infiltrated leaves, thereby mechanically inducing cell death. Each leaf disk was placed in 5 ml water, allowing for measurement of ion leakage with a conductivity meter. For mitogen-activated protein kinase (MAPK) assays, infiltrated leaves were snap frozen in liquid nitrogen 0, 5, 15, and 30 min after infiltration with 50 nM Kratos, Bia, or phosphate buffer.

Induction of cell death by oxidative stress using the so-called xanthine/xanthine oxidase (X/XO) system (Jabs *et al.*, 1996) has previously been described (Overmyer *et al.*, 2005). Briefly, fully expanded leaves of 5-day-old Col-0, *kratos-1*, and *bia-1* plants were detached and infiltrated with a buffer containing a superoxide-generating xanthine/xanthine oxidase mixture, or only buffer as a control. After 4 h, the detached leaves were rinsed and cell death from each leaf was quantified by placing the detached leaf in 5 ml water where ion leakage was measured with a conductivity meter, immediately (+0 h) or after another 4 h (+4 h).

### Reactive oxygen species burst measurements

Leaf discs were collected using a 4 mm cork borer from 4-week-old Arabidopsis Col-0 plants and floated overnight in sterile distilled water in a 96-well plate under continuous light at room temperature. On the following day, the water was replaced with assay buffer containing 34 mg l^−1^ Luminol sodium salt (Sigma), 20 mg l^−1^ horseradish peroxidase (Wako), 100 nM flg22 (GenScript), or synthetic peptides. Luminescence was measured using the GloMax®-Multi+Detection System (Promega). ROS production was expressed in relative luminescence units (RLU). Data are presented as the average of six leaves in a representative experiment and the experiment was repeated three times with similar results.

### Immunoblotting

Extracellular medium containing proteins >3 kDa after filtration was concentrated using Amicon centrifugal filter units (10 kDa cut-off, Millipore). The control cells were lysed in urea buffer (6 M urea, 50 mM Tris–HCl, pH 7.5, protease inhibitor cocktail 1× (Sigma-Aldrich)). Equal protein amounts (20–50 µg) separated by SDS-PAGE were transferred to polyvinylidene difluoride (PVDF) membranes (Bio-Rad). The membranes were blocked with 5% milk and probed with anti-HSP101 or anti-GDC-H antibody (1:1000 in 1% milk, TBS-T) (Agrisera). Horseradish peroxidase-conjugated donkey anti-rabbit IgG (GE Healthcare) was used as a secondary antibody and the signal was visualized by ECL Prime luminescence reagents (GE Healthcare).

For MAPK assay the frozen leaves were ground in liquid nitrogen to fine powder and the proteins were extracted by incubation for 30 min at 4 °C in extraction buffer (50 mM HEPES, pH 7.4, 50 mM NaCl, 10 mM EDTA, protease inhibitor cocktail (1×, Sigma-Aldrich), Halt phosphatase inhibitor cocktail (1×, Thermo Fisher Scientific)) with occasional vortexing. The supernatant after centrifugation at 16 000 *g* for 10 min at 4 °C was used for immunoblotting. One hundred micrograms of total protein was separated on 12% SDS-PAGE and transferred to PVDF membrane. The membrane was blocked with 5% milk in TBS and probed with phospho-p44/42 MAPK antibody (Cell Signaling Technology, no. 4370; 1:2000 in 1% milk, TBS-T). The signal was detected using fluorescent secondary antibody IRDye800CW Goat anti-Rabbit IgG (LI-COR, no. 926-32211) with an Odyssey fluorescence scanner (LI-COR Biosciences). Quantification of band intensity was performed with ImageJ.

### GAPDH cleavage analysis

GAPDH purified from rabbit muscle was purchased from Sigma-Aldrich (no. G5262, GAPDH standard for protein electrophoresis and no. G2267, GAPDH for enzymatic purposes). Recombinant Arabidopsis MC9 (rMC9) and mutated inactive rMC9^C/A^ (alanine substitution for the active site cysteine) fused to a His-tag were expressed and purified from *Escherichia coli* as previously described (Vercammen *et al.*, 2004). GAPDH proteins were dissolved at 2 mg ml^−1^. A dilution series of rMC9 in Milli-Q purified water was made starting from 1/25 (w/w) ratio. A 40 µl reaction mix consisted of 10 µl 4×MC9 buffer (200 mM MES pH 5.5, 600 mM NaCl, 40% sucrose, 0.4% CHAPS, and 40 mM DTT), 5 µl GAPDH protein, and 5 µl of rMC9 or rMC9^C/A^. rMC9 was added to the reaction mix first to auto-activate at room temperature. After 10 min, GAPDH protein was added and incubated for 30 min at 30 °C. The reaction was stopped by the addition of 10 µl 5× Laemmli buffer and samples were run on a 4–20% gradient SDS-PAGE gel (Mini-Protean TGX, Bio-Rad), stained with Instant Blue stain (Expedeon) and imaged with a ChemiDoc Imaging System (Bio-Rad).

## Results

### Quantitative peptidomics reveals extracellular peptides related to vascular xylem differentiation

**Fig. 1.**
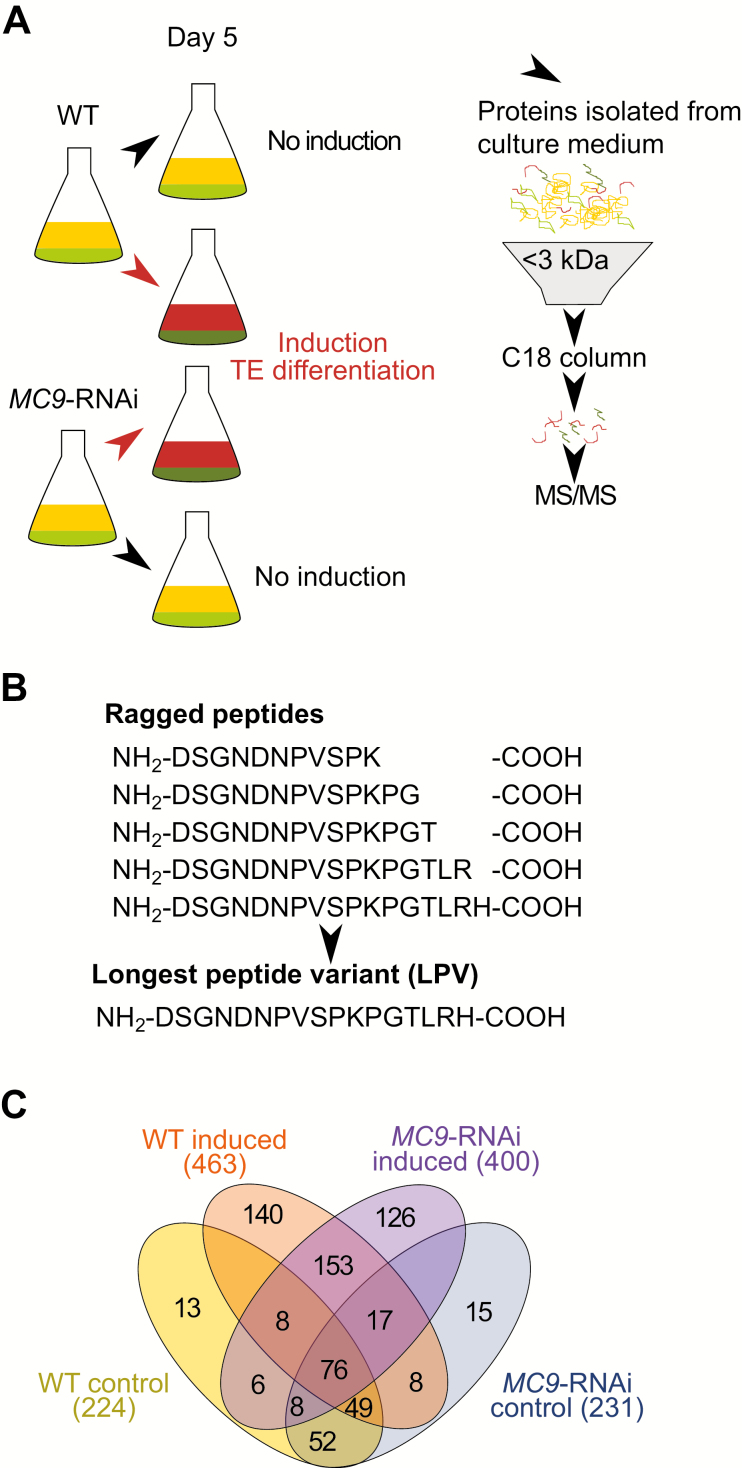
Peptidomics approach to identify METACASPASE9-regulated extracellular peptides. (A) Schematic representation of the bioactive peptide identification workflow. The extracellular media of differentiating cell suspensions were sampled 5 d after induction (*n*=3 biological replicates), half-way through the differentiation when *MC9* expression peaks and when ectopic cell death starts increasing in *MC9*-RNAi lines (Escamez *et al.*, 2016). (B) Visual explanation for the longest peptide variant (LPV) approach. (C) Venn diagram showing the number of unique peptides per genotype and per condition (induced or non-induced control).

To identify extracellular peptides in the context of vascular xylem differentiation, we used a cell suspension system in which hormonal induction triggers semi-synchronous vascular xylem differentiation over a period of 10 d (Pesquet *et al.*, 2010; Escamez *et al.*, 2016). Triplicate wild-type and *MC9*-down-regulated (*MC9*-RNAi) cell suspensions were induced to differentiate into xylem TEs and non-TEs, and cells were separated from the extracellular medium by filtration after 5 d when ectopic cell death starts increasing in *MC9*-RNAi lines (Escamez *et al.*, 2016). Control samples were collected from non-induced wild-type and *MC9*-RNAi cell suspensions. Proteins were isolated from the extracellular medium and peptides were separated from the higher molecular mass fraction by filtration over a 3 kDa cut-off filter (Fig. 1A). Immunoblotting of the higher molecular mass fraction (>3 kDa) showed no visible contamination with intracellular proteins (Supplementary Fig. S2A). The peptide-containing fraction (<3 kDa) was enriched and purified by solid phase extraction (SPE) (Fig. 1A). Subsequently, peptides were identified and quantified by LC-MS/MS using a Q Exactive mass spectrometer resulting in the identification of 1229 different peptides. Overlapping peptides matching to the same protein sequence, so-called ragged peptides, were assembled into the longest peptide variant (LPV; Fig. 1B) (Secher *et al.*, 2016). After LPV assembly, 722 unique peptides were identified, of which 689 were quantified in at least one replicate of one genotype in one condition (Supplementary Dataset S1).

The identified peptides showed a bias in amino acid composition, favouring Gly and Pro (Supplementary Fig. S2B). At the P1 position (position of the precursor protein sequence where cleavage releases the peptide), Gly or Lys was over-represented (Supplementary Fig. S2B). The fact that MC9 cleaves proteins after Arg or Lys (Tsiatsiani *et al.*, 2013) suggests that a subset of the peptides could result from MC9-mediated cleavage.

Induction of xylem differentiation doubled the peptidome repertoire in both wild-type and *MC9-*RNAi genotypes (Fig. 1C), likely reflecting an increase in proteolytic processes. Under control conditions the extracellular peptidomes of wild-type and *MC9*-RNAi largely overlapped (185 peptides, ~80% overlap; Fig. 1C), while they diverged more (254 peptides, ~60% overlap; Fig. 1C) after induction of xylem differentiation. Hence, xylem differentiation increased peptide diversity in a genotype-dependent manner. We reasoned that candidate regulator peptides for ectopic non-TE cell death should fulfil three criteria: (i) such peptides should accumulate during *in vitro* differentiation, (ii) they should be differentially abundant between wild-type (low ectopic death) and *MC9*-RNAi cells (elevated ectopic death), and (iii) the corresponding genes should be expressed during vascular differentiation. Based on these criteria, we prioritized a set of 15 candidate peptides for further characterization of their bioactivity (Supplementary Table S1).

### Kratos restricts runaway ectopic cell death during vascular differentiation

To test the effect of the candidate peptides on ectopic non-TE death, we used the VISUAL system (Kondo *et al.*, 2016) in which differentiation of vascular cells can be induced in the cotyledons (embryonic leaves) of Arabidopsis. This system enables differentiation of TEs within only 4 d (Kondo *et al.*, 2016), as confirmed by the expression patterns of TE marker genes (Bollhöner *et al.*, 2013; Escamez *et al.*, 2016), and by autofluorescence of lignin in the cell walls of TEs (Supplementary Fig. S3A–G). VISUAL also allows monitoring of non-TE cell death. Unlike TEs that become devoid of any visible content because they undergo complete protoplast autolysis (Escamez and Tuominen, 2017), ectopically dying non-TEs retain their protoplasts which become autofluorescent (Supplementary Fig. S3H–J). Autofluorescence of non-TE protoplasts was also observed following mechanical wounding of the cotyledons (Supplementary Fig. S3K–M), suggesting that protoplast autofluorescence could be used as a general hallmark of ectopic cell death in the VISUAL system.

**Fig. 2.**
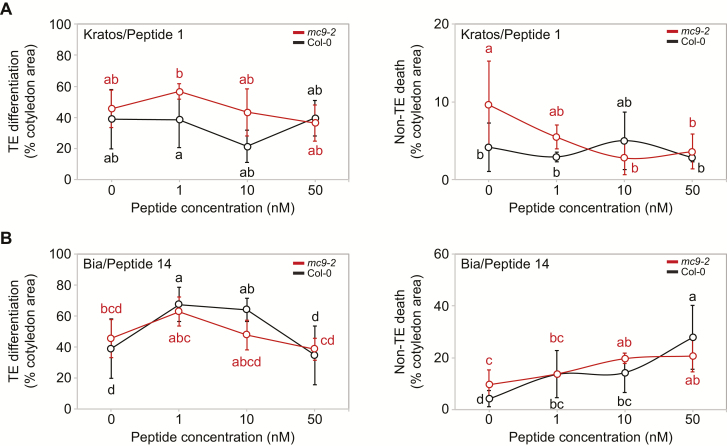
Unpurified peptides Kratos and Bia affect non-TE death in a dose-dependent manner. (A) TE differentiation (left) and non-TE death (right) 96 h after induction with the VISUAL method of Col-0 wild-type and *mc9-2* METACASPASE9 knockout mutant treated with increasing concentrations of Kratos/Peptide 1 (or only phosphate buffer as a control). Error bars represent standard deviation (*n*=3 biological replicates). Data points that do not share any letter are significantly different according to post-ANOVA Fisher’s test (*P*<0.05). (B) TE differentiation (left) and non-TE death (right) 96 h after induction with the VISUAL method of Col-0 wild-type and *mc9-2* METACASPASE9 knockout mutant treated with increasing concentrations of Bia/Peptide 14 (or only phosphate buffer as a control). Error bars represent standard deviation (*n*=3 biological replicates). Data points that do not share any letter are significantly different according to post-ANOVA Fisher’s test (*P*<0.05).

In a first screen, crude synthetic peptide solutions (i.e. without purifying the synthesized peptides) of the 15 candidate peptides were applied to the cotyledons of wild-type (Col-0) and MC9 knockout mutant *mc9-2* (Bollhöner *et al.*, 2013) seedlings 24 h after induction of vascular differentiation (Fig. 2A, B; Supplementary Fig. S4). At this stage the newly transdifferentiated pro-cambial cells start differentiating into other vascular cell types such as TEs (Kondo *et al.*, 2016). In agreement with previous results in *MC9*-RNAi cell suspensions (Escamez *et al.*, 2016), *mc9-2* mutants showed significantly more ectopic non-TE death than wild-type seedlings (Fig. 2A, B; Supplementary Fig. S4). Application of three peptides (Peptide 1, Peptides 3 and 4; Supplementary Table S1) led to a reduction of ectopic non-TE death in *mc9-2* to levels comparable to wild-type, without any dose-dependent effect on TE differentiation (Fig. 2A; Supplementary Fig. S4). Peptide 1, but not Peptide 3 or Peptide 4, reduced ectopic non-TE death in a dose-dependent manner (Fig. 2A). An opposite, dose-dependent increase in ectopic non-TE death was observed in both wild-type and *mc9-2* genotypes upon treatment with Peptide 14 (Fig. 2B).

**Fig. 3.**
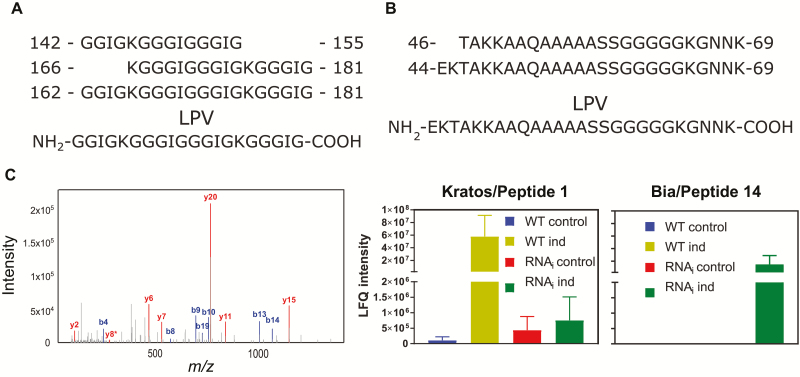
METACASPASE9-dependent accumulation of the peptides Kratos and Bia. (A) The assembly of the longest peptide variant (LPV) from the extracellular peptides matching the glycine-rich peptide Kratos/Peptide1 derived from the unknown protein AT3G23450 (Supplementary Table S1). (B) The assembly of the LPV from the extracellular peptides matching the small EDRK-rich factor (SERF) peptide Bia/Peptide 14 derived from the uncharacterized protein AT3G24100 (Supplementary Table S1). (C) Illustration of mass spectrometry identification of the longest identified peptide in (A) and charts displaying quantifications of the average label free quantification (LFQ) intensity for the LPV Kratos/Peptide 1 and Bia/Peptide 14 in the different genotypes and conditions. Error bars represent standard error of the mean (*n*=3 biological replicates).

In addition to its dose-dependent effect, the candidate Peptide 1 accumulated in the medium of wild-type cells, but not upon *MC9* down-regulation (Fig. 3A, C; Supplementary Dataset S1). Peptide 1 is part of a precursor protein of unknown function that contains a repeated motif of GGG(I/V)GGG(I/F)GK as well as six repeats of Peptide 1 (Supplementary Fig. S5A), suggesting a putative role as a precursor protein for bioactive peptides. The other peptide with a dose response, the candidate Peptide 14, accumulated only in the medium of differentiating *MC9*-RNAi cells (Fig. 3B, C). The precursor protein of Peptide 14 is part of an uncharacterized protein family founded by the highly conserved small EDRK-rich factor (SERF, previously known as H4F5) (Lefebvre *et al.*, 1995; Roy *et al.*, 1995; Kelter *et al.*, 2000) proteins in metazoans (Supplementary Fig. S5B, C). We named Peptide 1 (and its corresponding gene/protein AT3G23450) ‘Kratos’, and Peptide 14 (AT3G24100) ‘Bia’, in reference to the homonymous children of the Styx river separating the worlds of the living and of the dead in the ancient Greek mythology.

**Fig. 4.**
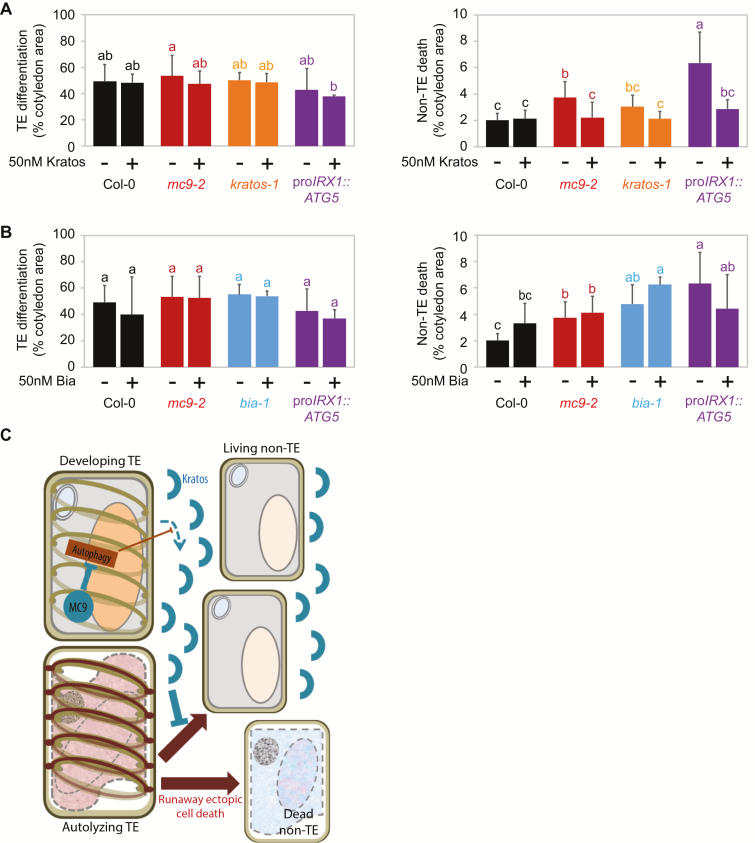
Ectopic non-TE death is restricted by the Kratos peptide during induced vascular differentiation in cotyledons. (A) TE differentiation (left) and non-TE death (right) 96 h after induction with the VISUAL method of Col-0, *mc9-2*, *kratos-1* knockout mutant and pro*IRX1::ATG5* TE autophagy inducer line with or without 50 nM Kratos. Error bars represent standard deviation (*n*=9 replicate experiments for Col-0 and *mc9-2*, 5 for Col-0+Kratos, *mc9-2*+Kratos, *kratos-1* and *kratos-1*+Kratos, 4 for pro*IRX1::ATG5*, and 3 for pro*IRX1::ATG5*+Kratos, each with three biological replicates). Data points that do not share any letter are significantly different according to post-ANOVA Fisher’s test (*P*<0.05). (B) TE differentiation (left) and non-TE death (right) 96 h after induction with the VISUAL method of Col-0, mc9-2, *bia-1* knockout mutant and pro*IRX1::ATG5* TE autophagy inducer line with or without 50 nM Bia. Error bars represent standard deviation (*n*=9 replicate experiments for Col-0 and *mc9-2*, 8 for Col-0+Bia and *mc9-2*+Bia, 4 for pro*IRX1::ATG5*, and 3 for *bia-1*, *bia-1*+Bia and pro*IRX1::ATG5*+Bia, each with three biological replicates). Data points that do not share any letter are significantly different according to post-ANOVA Fisher’s test (*P*<0.05). (C) Hypothetical model for the modulation of non-TE ectopic death by MC9 and autophagy in TEs through regulation of extracellular accumulation of the Kratos peptide.

To confirm the cell-survival effect of Kratos, Peptide 3, and Peptide 4, as well as the death-promoting effect of Bia, we repeated the treatments of differentiating cells in the VISUAL system with purified synthetic peptides (purity >95%). Treatment with purified Kratos confirmed its ability to decrease ectopic non-TE death in *mc9-2* without affecting TE differentiation (Fig. 4A). Furthermore, a knockout T-DNA line for the Kratos-encoding gene (*AT3G23450*; *kratos-1*)—devoid of visible xylem differentiation defect (Supplementary Fig. S6)—showed a trend towards higher ectopic non-TE death than wild-type (Fig. 4A), which could be complemented by treatment with purified Kratos (Fig. 4A). These results, together with the reduced abundance of extracellular Kratos in differentiating *MC9*-RNAi cell suspensions (Fig. 3C) and the increased ectopic non-TE death upon loss of MC9 function (Figs 2, 4), indicate that Kratos functions in restricting MC9-dependent ectopic non-TE death during xylem differentiation.

Peptides 3 and 4 were disqualified as potential regulators of ectopic non-TE death based on the inability of the corresponding purified peptides to significantly decrease ectopic non-TE death in *mc9-2* (Supplementary Fig. S7), and because T-DNA lines for the corresponding genes did not display consistent changes in ectopic death (Supplementary Fig. S7). The potential role of Bia in promoting ectopic non-TE death during vascular differentiation remained unclear because treatment with the corresponding purified peptides produced inconsistent effects depending on the treated genotype (Fig. 4B). Furthermore, even without treatment ectopic non-TE death tended to increase, rather than decrease, in a T-DNA knockout line for the Bia-encoding gene (*AT3G24100*; *bia-1*; Fig. 4B).

### Kratos functions downstream of autophagy in TEs

Autophagy has been suggested to increase in the TEs of *MC9*-down-regulated cell suspensions, which also display elevated ectopic non-TE death (Escamez *et al.*, 2016). Cell-type-specific down-regulation of the autophagy gene *ATG2* to decrease autophagy in TEs was found sufficient to restrict ectopic non-TE death in *MC9*-down-regulated cell suspensions (Escamez *et al.*, 2016). Consistent with the modulation of autophagy by MC9 in TEs, we found a partial co-localization between MC9 and the vascularly expressed autophagosome marker ATG8a (Furuta *et al.*, 2014) in TEs in roots of Arabidopsis seedlings (Supplementary Fig. S8A). Visualization of autophagosomes with the same marker showed increased autophagy in the TEs of *mc9-2* compared with the wild-type (Supplementary Fig. S8B). Furthermore, the extracellular medium of xylem differentiating cell suspensions contained six unique peptides matching three subunits of the GAPDH protein (Supplementary Dataset S1), a known regulator of autophagy (Colell *et al.*, 2007; Tristan *et al.*, 2011; Henry *et al.*, 2015), as well as an evolutionarily conserved target of MC9 and its orthologues (Tsiatsiani *et al.*, 2013). Five of the six peptides from GAPDH precursor proteins contain potential MC9 cleavage sites (Supplementary Fig. S9A). Mammalian GAPDH could be cleaved *in vitro* by recombinant Arabidopsis MC9 in a dose-dependent manner, while no cleavage was observed in the absence of MC9 or in the presence of catalytically inactive MC9 (Supplementary Fig. S9B). Sequence alignment between the assayed mammalian GAPDH and the Arabidopsis GAPDH proteins showed a high degree of conservation, including conservation of three potential MC9 cleavage sites (Supplementary Fig. S9C) between the detected Arabidopsis GAPDH-derived peptides (Supplementary Fig. S9A) and the assayed mammalian protein (Supplementary Fig. S9C). It is therefore reasonable to hypothesize that MC9 regulates autophagy in TEs by cleaving GAPDH, which would also lead to the release of GAPDH peptides during TE autolysis, thereby explaining that we detected extracellular GAPDH-derived peptides.

Given that TE autophagy modulates ectopic non-TE death downstream of MC9, it is possible that modulation of autophagy in TEs could function upstream of the Kratos-mediated restriction of ectopic non-TE death. To test this hypothesis, we increased the level of autophagy in TEs by overexpressing the autophagy rate-limiting protein ATG5 (Minina *et al.*, 2018) under the transcriptional control of the *IRX1* promoter (pro*IRX1::ATG5*), which is active in TEs and not in non-TEs (Escamez *et al.*, 2016) (Supplementary Fig. S3A–G). Overexpression of ATG5 in TEs increased autophagy in these cells (Supplementary Fig. S8C) without significant effect on TE differentiation compared with wild-type (Fig. 4A, B). Instead, consistent with a link between TE autophagy and ectopic non-TE death, ATG5 overexpression in TEs resulted in a significant increase in ectopic non-TE death (Fig. 4A, B). Ectopic non-TE death in pro*IRX1::ATG5* cotyledons was decreased to wild-type levels by treatment with Kratos peptide (Fig. 4A). Given that extracellular abundance of Kratos was reduced in cell suspensions where TEs displayed MC9 down-regulation and hence increased autophagy (Fig. 3C), the restriction of ectopic non-TE death in pro*IRX1::ATG5* by Kratos (Fig. 4A) suggests that this peptide functions downstream of TE autophagy (Fig. 4C). Unexpectedly, ectopic non-TE death in pro*IRX1::ATG5* was decreased upon treatment with Bia (Fig. 4B), suggesting that the relation between Bia and TE autophagy may be complex.

### Kratos and Bia modulate ectopic cell death induced by stress

The ability of Kratos (Figs 2A, 4A) to modulate ectopic cell death during induction of vascular differentiation, and the possible death-promoting effect of Bia (Fig. 2B), prompted us to test whether these peptides could modulate ectopic cell death in other contexts than vascular differentiation.

**Fig. 5.**
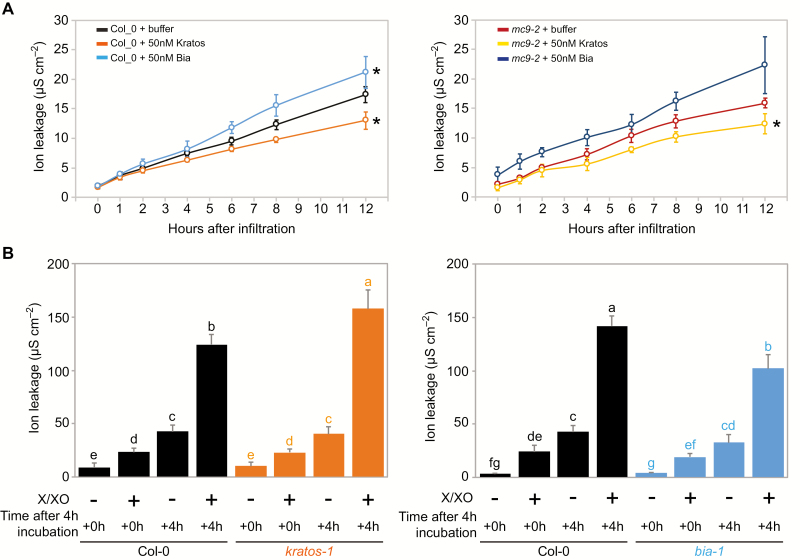
Peptides Kratos and Bia have the ability to modulate stress-induced cell death. (A) Mechanically induced cell death (measured by ion leakage) in leaf disks from Col-0 wild-type or *mc9-2* leaves infiltrated with Kratos or Bia peptides, or with buffer as a control. Data points indicate average values while error bars represent standard deviation (*n*=4 biological replicates). Asterisks indicate significantly different (*P*<0.05) ion leakage dynamics compared with buffer control. (B) Cell death induced by 4 h treatment with superoxide-generating xanthine/xanthine oxidase (X/XO) and quantified by measuring ion leakage in detached leaves from Col-0, *kratos-1*, and *bia-1* 0 h (+0 h) or 4 h (+4 h) after the end of the 4 h treatment. Bars indicate average values while error bars represent standard deviation (*n*=4 biological replicates). Data points that do not share any letter are significantly different according to post-ANOVA Fisher’s test (*P*<0.05). The experiments have been repeated twice with similar results.

Mechanical stress was imposed to induce wounding and subsequent ectopic cell death by excision of leaf disks from wild-type and *mc9-2* leaves and infiltration with buffer alone or with peptide. Indeed, following local mechanical stress, cell death has been shown to spread from the site of application to other cells (Greenberg and Ausubel, 1993; Wrzaczek *et al.*, 2015). Here, cell death was estimated over time by measuring electrolyte leakage (Fig. 5A). Electrolyte leakage dynamics were comparable between wild-type and *mc9-2* (Fig. 5A), as expected from the fact that *MC9* is expressed in cells undergoing developmental, rather than accidental, cell death (Olvera-Carrillo *et al.*, 2015). Cell death increased in both genotypes upon treatment with the Bia peptide (Fig. 5A), consistent with an ectopic cell death-promoting role. Treatment with the Kratos peptide decreased cell death following mechanical stress regardless of the genotype (Fig. 5A), consistent with the ability of Kratos to restrict ectopic cell death.

Additionally, cell death was induced by extracellular reactive oxygen species (ROS) production in leaves of wild-type as well as in the null mutants *kratos-1* and *bia-1*, and cell death was measured by electrolyte leakage after 4 h (Fig. 5B). Cell death in response to extracellular ROS production was significantly higher in *kratos-1* plants compared with wild-type (Fig. 5B), consistent with an anti-ectopic cell death role for Kratos. The ability of Bia to enhance cell death was also supported by the fact that *bia-1* plants displayed less ectopic cell death than wild-type plants (Fig. 5B).

Kratos and Bia regulated cell death in ways that are reminiscent of responses to microbe-associated molecular patters (MAMPs) and/or damage-associated molecular patterns (DAMPs) (Bigeard *et al.*, 2015). MAMPs and DAMPs trigger signalling cascades whose hallmarks include the production of extracellular ROS and activation of mitogen-activated protein kinases (MAPK or MPK), especially MPK3 and MPK6 (Bigeard *et al.*, 2015). We investigated whether Kratos and Bia could modulate ectopic cell death through similar signalling networks. Unlike the MAMP flg22, an epitope of the bacterial flagella which triggers an oxidative burst, neither Kratos nor Bia application triggered a ROS burst (Supplementary Fig. S10). On the other hand, MPK3 and MPK6 activation, which typically peaks within 10–15 min after signal perception (Bigeard *et al.*, 2015), was higher 15 min after infiltration of Arabidopsis leaves with Kratos or Bia compared with controls (Supplementary Fig. S10). Together these results suggest that some downstream responses to Kratos and Bia could be shared with MAMPs or DAMPs while others seem to be distinct.

Our collective results reveal that Kratos has the ability to restrict ectopic cell death both in a normal developmental context and as a consequence of stress, while Bia conclusively promotes ectopic cell death only in the tested stress conditions. At least during xylem differentiation and wounding, cell death can spread in a regulated manner, which reveals that the original trigger itself does not necessarily cause all of the observed death events. Instead, the cell death that ectopically spreads likely represents a specific type of cell death with its own genetic regulation (Wrzaczek *et al.*, 2009; Wrzaczek *et al.*, 2015) (Figs 4A, 5) that relies on extracellular peptides (Figs 3–5), one of which apparently functions downstream of autophagy in TEs (Fig. 4A).

## Discussion

By combining peptidomics with the use of a controlled plant vascular differentiation system, we identified 1229 extracellular peptides, corresponding to 433 proteins, in the context of plant vascular development (Supplementary Dataset S1). These numbers, as often with peptidomics, differ from current high mass-accuracy shotgun proteomics. Nevertheless, our analysis performed at least as well as other label-free peptidomics (Nanni *et al.*, 2009; Ling *et al.*, 2010; Chen *et al.*, 2014) studies when considering the caveats associated with endogenous peptidomics (Dallas *et al.*, 2015). One frequently reported challenge in peptidomics is the occurrence of progressively shortened N- or C-terminal peptide ends. Assuming that they belong to the same (bioactive) precursor, we aggregated such ragged peptides to a single longest peptide variant (LPV; Fig. 1B), thereby facilitating data interpretation and enhancing label-free quantification as the intensity of the LPVs is the sum of the intensities of all their nested peptides. A previous proteomics study identified 149 proteins with an orthologue in Arabidopsis in the xylem sap of *Brassica oleracea* (Ligat *et al.*, 2011) but did not focus on the peptide fraction. While the different methods and data processing pipelines between both studies render direct comparison of the detected peptides challenging (Ligat *et al.*, 2011), 27 proteins identified in the xylem sap of *Brassica oleracea* matched precursor proteins of the peptides identified in our dataset (Supplementary Dataset S1). This suggests that our dataset is relevant for the developmental context of vascular xylem differentiation.

Differentiating TEs undergo developmentally regulated cell death and protoplast autolysis (Escamez and Tuominen, 2014), during which they release proteases, and likely other molecules, which can either directly injure and kill neighbouring cells (Endo *et al.*, 2001) or possibly activate signalling towards ectopic cell death (Endo *et al.*, 2001; Escamez *et al.*, 2016). To prevent ectopic non-TE death, it was shown that at least one protease inhibitor must be released in the extracellular medium of xylem-differentiating cell suspensions in *Zinnia elegans* (Endo *et al.*, 2001). We report here the extracellular peptide Kratos as another factor that is required to restrict ectopic non-TE death in Arabidopsis. Kratos was abundant in wild-type xylem-differentiating cell suspensions (Fig. 3C), and it rescued the ectopic cell death during vascular differentiation in the *mc9-2* mutant (Figs 2A, 4A). Another peptide, Bia, was only detected in the extracellular medium of differentiating, ectopic cell death-prone *MC9*-RNAi cell suspensions (Fig. 3C) and displayed a tendency to promote ectopic cell death following stress (Fig. 5A, B). Our data suggests that ectopic non-TE death is modulated by a balance between pro-death and pro-survival factors. This balance is altered when *MC9* is down-regulated (Fig. 3C; Supplementary Dataset S1), resulting in enhanced ectopic death (Escamez *et al.*, 2016) (Figs 4, 5). We identified the extracellular peptide Kratos as one of these factors regulating runaway ectopic cell death, and possibly also Bia. Neither of the precursor proteins for Bia or Kratos had been found among MC9 targets in Arabidopsis seedlings and their cleavage sites do not display the MC9-specific lysine or arginine amino acid at the P1 position. Hence, Kratos and Bia are likely indirect targets of MC9, possibly through MC9-dependent modulation of TE autophagy (Supplementary Fig. S8A, B) (Escamez *et al.*, 2016), as seems to be the case for Kratos (Figs 3C; 4A).

An important function of MC9 in TEs during vascular development is the regulation of autophagy (Escamez *et al.*, 2016) (Supplementary Fig. S8B), which in turn regulates non-TE ectopic cell death (Escamez *et al.*, 2016) (Fig. 4A–C). More precisely, our results indicate that MC9 tunes down the level of autophagy in TEs, while TE autophagy negatively regulates the extracellular accumulation of the ectopic cell death-restricting Kratos peptide (Fig. 4C). Frequent identification of peptides derived from GAPDH (Supplementary Dataset S1), which is known to regulate autophagy (Colell *et al.*, 2007; Tristan *et al.*, 2011; Henry *et al.*, 2015) and can be cleaved by MC9 (Tsiatsiani *et al.*, 2013) (Supplementary Fig. S9B), allows us to hypothesize that GAPDH cleavage by MC9 could mediate the modulation of autophagy upstream of ectopic cell death.

Autophagy is known as a cellular process in which double-membrane-enclosed autophagosome vesicles transport cellular components to vacuoles or lysosomes for degradation and recycling (Marshall and Vierstra, 2018), which seems contradictory to a role in regulating accumulation of extracellular peptides.   Yet, studies in animals have found a role for autophagy in secreting proteins or peptides (Bhattacharya *et al.*, 2014; Kimura *et al.*, 2017*b*), which has also been hypothesized in plants (Pečenková *et al.*, 2017). Hence, TE autophagy may directly regulate extracellular peptide accumulation similar to the secretion of the mammalian pro-inflammatory interleukin-1β peptide by autophagy (Dupont *et al.*, 2011). TE autophagy may also play a role in degrading an upstream regulator of extracellular peptide accumulation, as observed for mammalian senescence-associated secretory phenotype (Kang *et al.*, 2015). A third possibility is that different levels of autophagy in TEs could influence the intracellular content released by TEs upon loss of plasma membrane integrity during developmental cell death and autolysis.

Intriguingly, while identified in a specific developmental context, both Kratos and Bia possess the ability to modulate ectopic cell death that results from stress. Stress-induced cell death and developmental cell death are known to display distinct transcriptional and anatomical hallmarks (Overmyer *et al.*, 2005; Torres *et al.*, 2005; van Doorn, 2011; Fendrych *et al.*, 2014; Olvera-Carrillo *et al.*, 2015; Escamez and Tuominen, 2017), indicating that they represent different types of cell death. It is therefore surprising to find that Kratos and Bia can modulate ectopic cell death occurrences that follow different triggers. A possible explanation is that, after stress-induced or developmental cell death, the resulting ectopic cell death represents one specific type of cell death with its own anatomical features and molecular machinery, different from the cell death that is induced directly in response to the initial stimulus. This seems to be the case at least for the ectopic non-TE death that is anatomically distinct from the cell death of the TEs (Escamez *et al.*, 2016) (Supplementary Fig. S3H–M), and where only the former and not the latter is affected by Kratos (Escamez *et al.*, 2016) (Figs 2A, 4A). The spreading, runaway cell death in plants that follows stress-induced or developmental cell death may therefore be analogous to an inflammation-like response caused by the initial, ‘primary’ cell death. Given our finding that Kratos can restrict runaway ectopic cell death in developmental and stress-related contexts, we hypothesize the existence of a plant inflammation-like response involving Kratos as a canonical effector.

## Supplementary data

Dataset S1. Peptide identification from extracellular medium of cell suspensions differentiating (or not as a control) into a mixture of xylem tracheary elements (TE) and non-TE cells.

Fig. S1. Identification of knockout T-DNA lines for Kratos and Bia.

Fig. S2. Quantitative peptidomics on extracellular medium of xylogenic Arabidopsis cell suspensions.

Fig. S3. Monitoring TE differentiation and non-TE death with the Vascular Cell Induction Culture System Using Arabidopsis Leaves (VISUAL).

Fig. S4. Effect of candidate peptides from crude extracts on TE dierentiation and non-TE death.

Fig. S5. Sequences of the precursor proteins from which the peptides Kratos and Bia are generated.

Fig. S6. Normal vascular development in the cotyledons of the *mc9-2*, *bia-1*, and *kratos-1* mutants.

Fig. S7. Peptides 3 and 4 do not affect non-TE death.

Fig. S8. METACASPASE9 and autophagy are linked in TEs.

Fig. S9. GAPDH peptides are detected in extracellular peptidomics of differentiating cells, possible from cleavage of GAPDH by MC9.

Fig. S10. Kratos and Bia do not trigger oxidative burst.

Table S1. List of the unique peptides selected for analyses during vascular differentiation.

Supplementary Dataset S1

Supplementary Figures S1-S10

Supplementary Table S1

## Author contributions

SE, SS, JPV, MW, and HT conceived the study and obtained or generated the plant material and peptides. SE generated material from cell suspensions for peptide identification. SS, JPV, FVB, and KG performed peptide isolation, enrichment, identification, and data interpretation. SE performed all experiments of xylem differentiation. JPV, HJ, SK, and MW performed stress-induced cell death assays, MPK activity assays, and ROS measurements. SE performed confocal laser scanning microscopy analyses. SE, SS, MW, KG, and HT wrote the manuscript with help from all the other authors.
