# Supplementary material for: Extracellular peptide Kratos restricts cell death during vascular development and stress in Arabidopsis
Source: J Exp Bot. 2019 Feb 7;70(7):2199–210. doi: 10.1093/jxb/erz021 (PMC6460963; doi:10.1093/jxb/erz021)
Supplement: Supplementary Figures S1-S10 [file erz021_suppl_supplementary_figures-s1-s10.pdf]

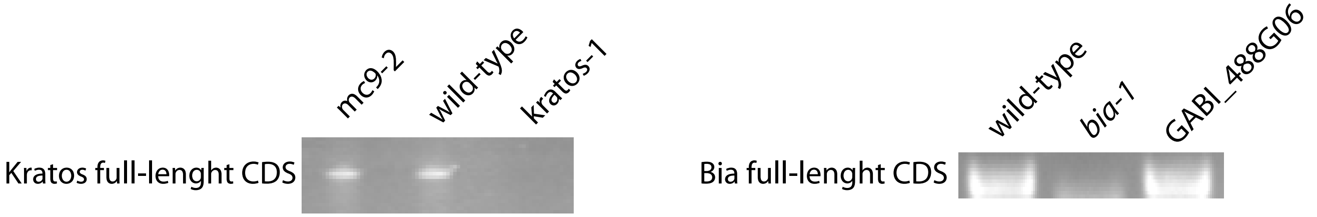

Figure S1: Identification of knockout T-DNA lines for Kratos and Bia
Semi-quantitative PCR comparing transcript levels (full length coding sequence: CDS) of Kratos and Bia between T-DNA lines for these genes (*kratos-1* and *bia-1*, respectively) and wild-type (and *mc9-2* for Kratos). At least three plants of each genotype have been pooled before RNA isolation.

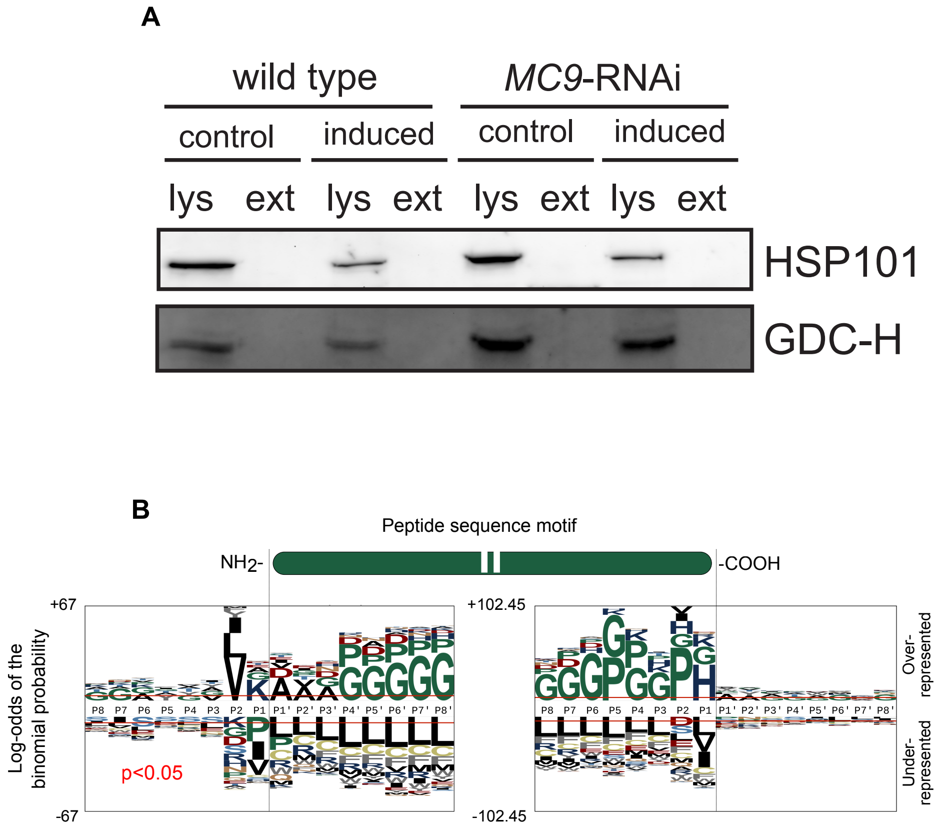

Figure S2: Quantitative peptidomics on extracellular medium of xylogenic *Arabidopsis* cell suspensions.
(A) Westerm blot analysis of cell lysate (lys) amd extracellular medium (ext) fractions after separation by filtration, to control for possible contamination of the extracellular fraction by intracellular proteins. HSP101 is a cytosolic heat shock protein (101kDa). GDC-H is a mitochondrial glycine decarboxylase complex subunit.
(B) Peptide sequence motif for all 1229 identified peptides generated by pLogo, which visualizes the probability of occurrence of a certain amino acid in a certain position relative to the amino acid distribution of the *Arabidopsis* proteome. The left box visualizes the 8 amino acids surrounding the N-terminal end of the endogenous peptides (labeled P8 till P1 and P1′ till P8′), while the right box visualizes those surrounding the C-terminus. This analysis was performed using pLogo (O'Shea JP, Chou MF, Quader SA, Ryan JK, Church GM, & Schwartz D. 2013. pLogo: A probabilistic approach to visualizing sequence motifs. Nat Methods 10, 1211-1212.).

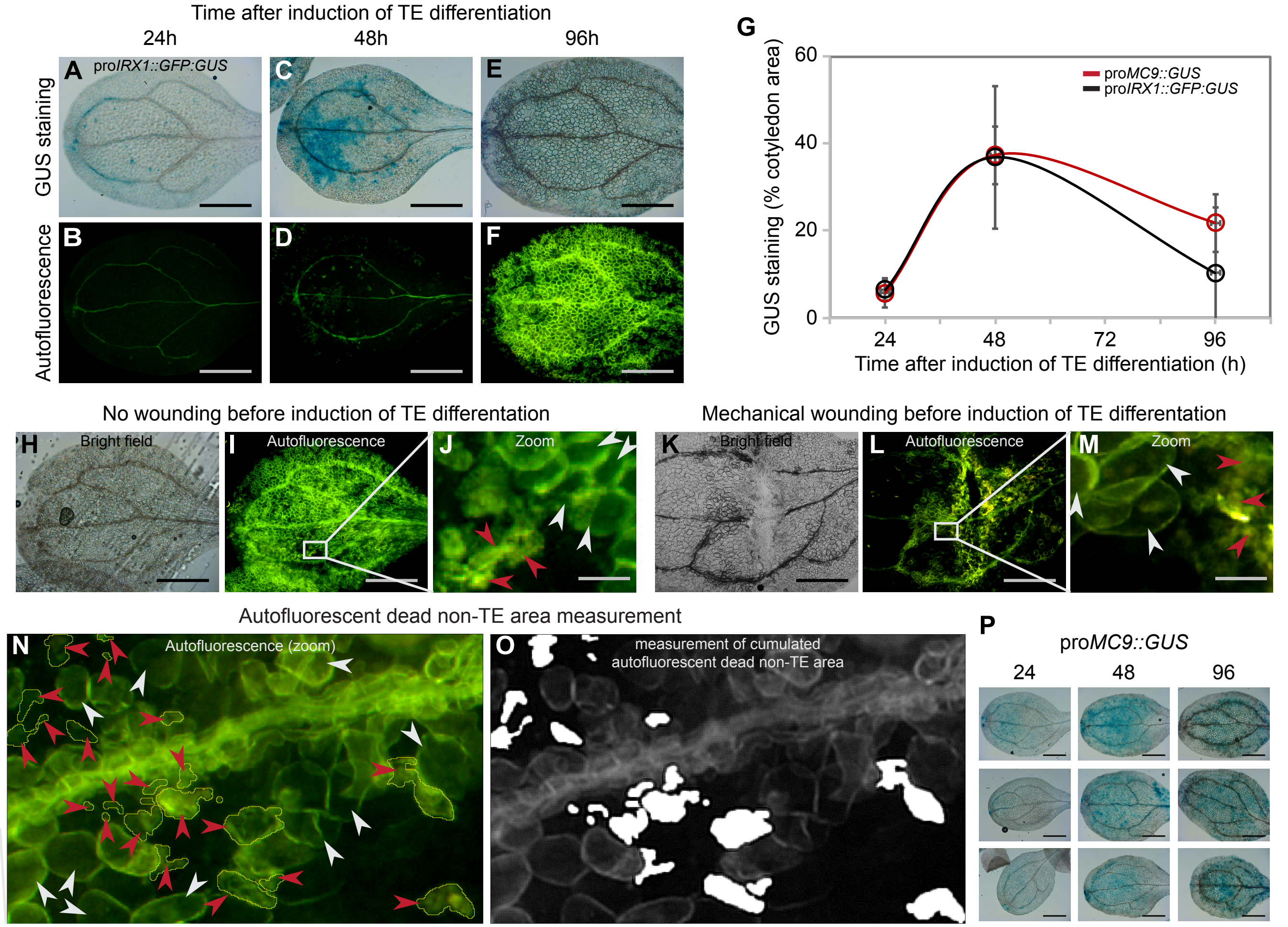

Figure S3: Monitoring TE differentiation and non-TE death with the Vascular Cell Induction Culture System Using Arabidopsis Leaves (VISUAL).

(A-F) Micrographs of Arabidopsis pro*IRX1::GFP:GUS* (11) cotyledons induced for TE differentiation using the VISUAL method (28). (A, C, and E) display GUS staining (blue) at different time points after induction of differention. The GUS staining reports activity of the *IRX1* promoter which is specific to the tracheary element (TE) cell type in this experimental system. The disapearance of GUS staining at the last time point is due to the developmental cell death and autolysis of the TE cell type. (B, D, and E) show autofluorescence originating mainly from lignin in the cell walls of the TE cell type, which increases over time as more TEs finish their differentiation. Scale bars = 1 mm.

(G) Quantification of GUS signal over the course of TE differentiation in the cotyledons of the pro*MC9::GUS* and pro*IRX1::GFP:GUS* reporter lines. The GUS signal increases between 24h and 48h, as expected from the fact that TE cells start differentiating (ref. 35), while the GUS staining strongly decreases at 96h as expected from the fact that TEs undergo developmental cell death. Error bars represent standard deviation (n = 3 biological replicates).

(H-M) Micrographs of Col-0 wild-type cotyledons 96h after induction TE differentiation with the VISUAL methods without (H-J) or after (K-M) mechanical wounding. Ectopic non-TE cell death that occurs as a consequence of wounding (M) or as a consequence of TE differentiation (J) is revealed by shrunken, autofluorescent protoplasts (red arrowheads) which are absent from fully autolyzed, hollowed TE cells (white arrowheads) whose cell walls are autofluorescing. Scale bars = 1 mm in the full cotyledons micrographs and 100 μm in the corresponding zoomed images.

(N,O) Illustration of the quantification process of dead non-TE areas of induced cotyledons based on autofluorescence micrographs. (N) The cells displaying features of dead non-TEs (red arrows) such as shrunken autofluorescent shrunken protoplast clearly different from empty TEs that only show cell wall autofluorescence (white arrows) were selected using the "selection brush tool" in ImageJ and there cumulated area over the entire cotyledon was measured as illustrated in (O).

(P) Micrographs of GUS histochemical staining for pro*MC9::GUS* quantified in (g). Remainder GUS signal at 96h is localized in cells with visible cell wall thickenings which indicate that they are tracheary elements. Bars = 1mm

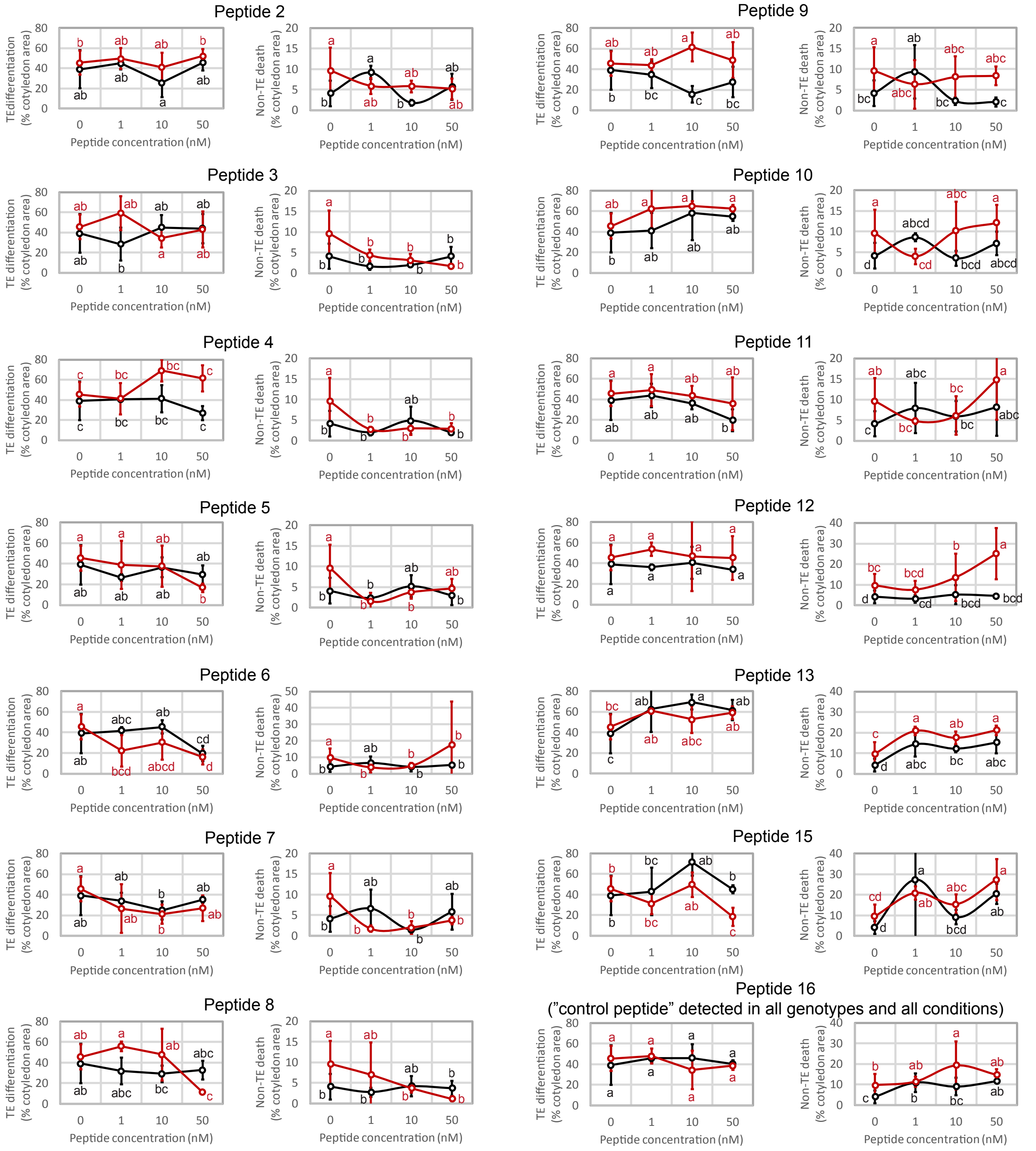

Figure S4: Effect of candidate peptides from crude extracts on TE differentiation and non-TE death
TE differentiation (left) and non-TE death (right) 96h after induction with the VISUAL method of Col-0 wild-type and *mc9-2* METACASPASE9 knock-out mutant treated with increasing concentrations of indicated peptide (or only phosphate buffer as a conctrol). Error bars represent standard deviation (n = 3 biological replicates). Data points that do not share any letter are significantly different according to post-ANOVA Fischer test ($p < 0.05$).

A

```
1   MGRLVSGATL LALLCFHVFV VNVVARDVSS GRDEDEKTLV GGGKGGGFGG
51  GFGGGAGGGV GGGAGGGFGG GAGGGFGGGG GGGGGGGGGG GGGFGGGGGF
101 GGGHGGGVGG GVGGGHGGGV GGGFGKGGGI GGGIGKGGGV GGGIGKGGGI
151 GGGIGKGGGV GGGIGKGGGI GGGIGKGGGI GGGIGKGGGI GGGIGKGGGI
201 GGGIGKGGGV GGGFGKGGGV GGGIGKGGGV GGGFGKGGGV GGGIGKGGGI
251 GGGIGKGGGI GGGIGKGGGI GGGIGKGGGI GGGIGKGGGI GGGIGKGGGI
301 GGGIGKGGGI GGGIGKGGGI GGGGGFGKGG GIGGGIGKGG GIGGGGGFGK
351 GGGIGGGIGK GGGIGGGFGK GGGIGGGIGG GGGFGGGGGF GKGGGIGGGI
401 GKGGGFGGGG GFGKGGGIGG GGGFGKGGGF GGGGFGGGGG GGGGGGGGIG
451 HH
```

Sequence repeats
GGGIGGGIGK * 16
GGGVGGGIGK * 4
GGGIGGGFGK * 3

Predicted signal peptide
Predicted transmembrane domain

B

```
1  MTRGSQRERD RERALARTGG KGKNKDDGLT PEQRRERDAK ALQEKTAKKA
51 AQAAAAASSG GGGGKGNNK
```

Predicted:
Nucleus or mitochondrial

C

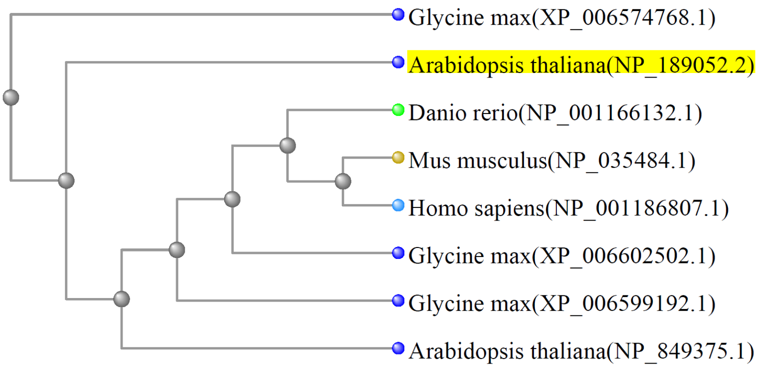

Figure S5: Sequences of the precursor proteins from which the peptides Kratos and Bia are generated.
(A) Sequence of the protein from which the Kratos peptide is generated. Predicted motifs based on protein sequence are highlighted with colors and described below the sequence, while the Kratos peptide is underlined.
(B) Sequence of the protein from which the Bia peptide is generated. Predicted localization based on protein sequence is indicated below the sequence, while the Bia peptide is underlined.
(C) Phylogenetic tree of SERF family proteins, using the precursor protein from Bia/Peptide 14 as query (highlighted) for protein BLAST in NCBI, against the "Model Organisms" database. Multiple Protein alignment and phylogenetic tree were generated using COBALT (Papadopoulos JS and Agarwala R, 2007. Bioinformatics 23:1073-79) using the "Fast Minimum Evolution" method.

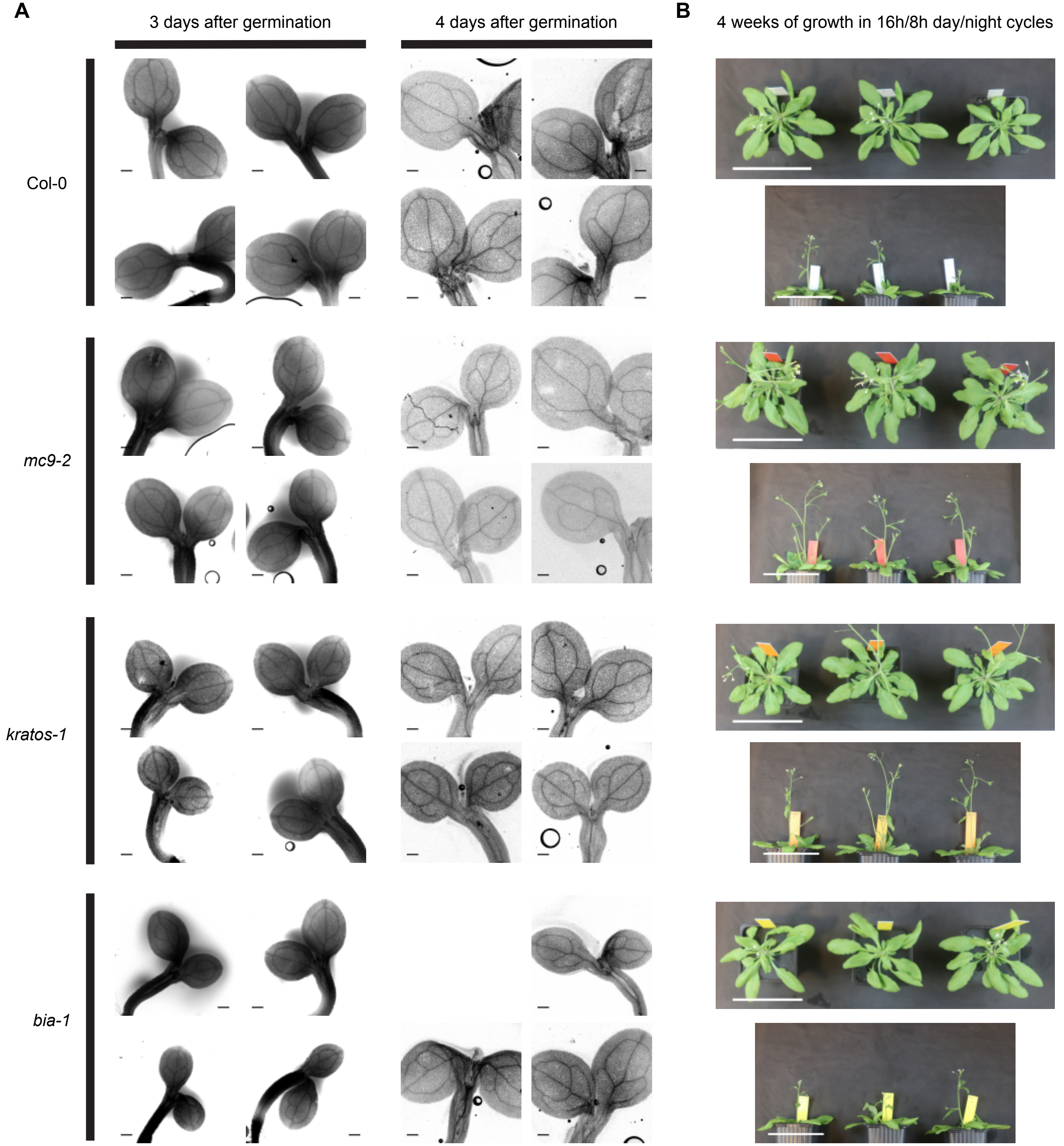

Figure S6: Normal vascular development in the cotyledons of the *mc9-2*, *bia-1* and *kratos-1* mutants.
(A) Micrographs of the Col-0, *mc9-2*, *bia-1* and *kratos-1* seedlings stained with Evans blue (stains mostly dead vascular tracheray elements, used here to highlight vasculature), imaged 3 and 4 days after germination. Bars = 200 µm.
(B) Images showing 4-weeks-old plants of the Col-0, *mc9-2*, *bia-1* and *kratos-1* genotypes grown in standard greenhouse conditions (16h/8h day/night cycle, and18°C < temperature < 23°C). Bars = 10cm
The pattern of vasculature (clearly visible from the Evans blue staining of dead vascular TEs) did not appear to clearly differ between the genotypes and the apparently normal growth of all mutants suggest normal vascular development, at least in optimal growth conditions.

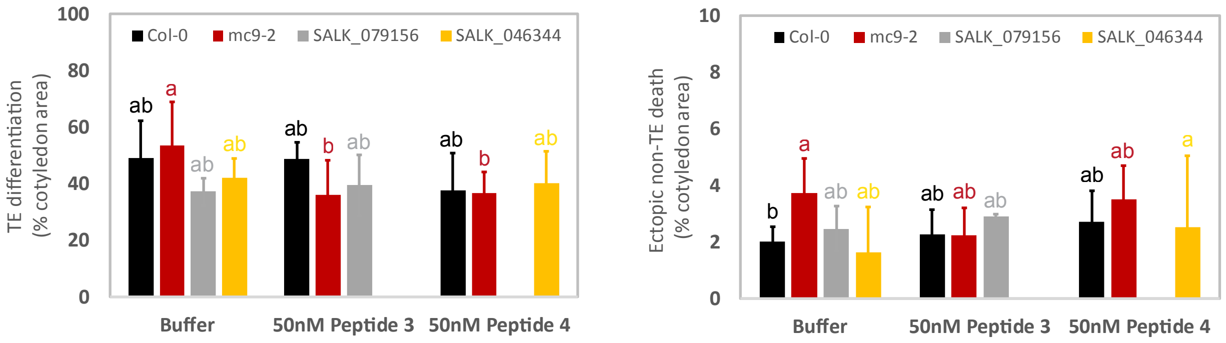

Figure S7: Peptides 3 and 4 do not affect non-TE death.
TE differentiation (left) and non-TE death (right) 96h after induction with the VISUAL method of Col-0, *mc9-2*, peptide 3 knock-out mutant (SALK_079156) and peptide 4 knock-out mutant (SALK_046344) treated or not with 50 nM Peptide. Error bars represent standard deviation (n = 3-9 replicate experiments). Data points that do not share any letter are significantly different according to post-ANOVA Fischer test ($p < 0.05$).

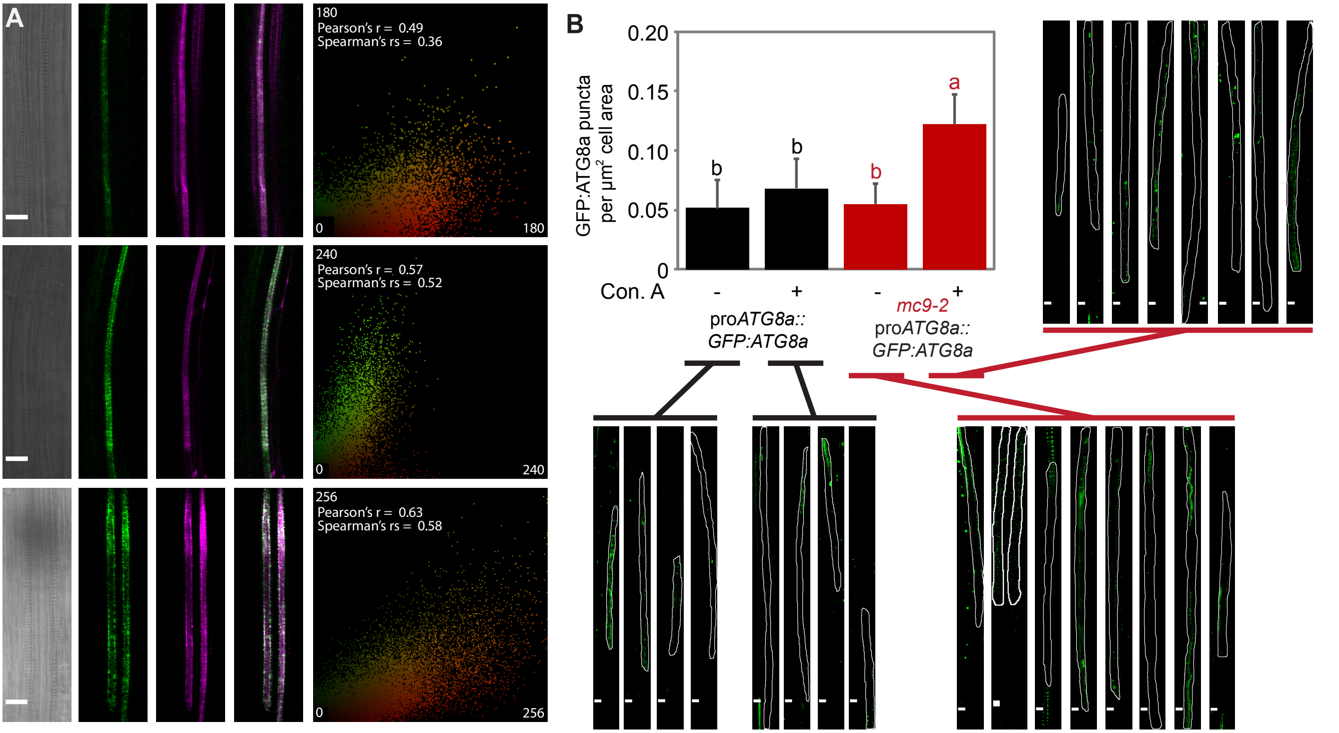

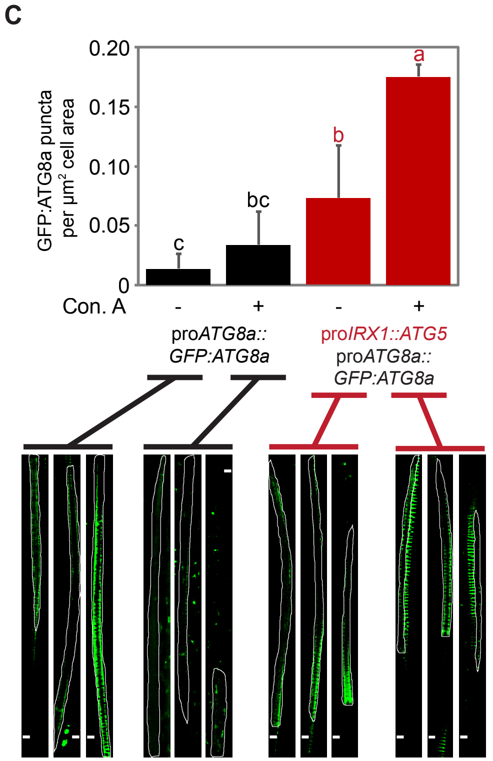

Figure S8: METACASPASE9 and autophagy are linked in TEs.
(A) Confocal Laser Scanning Microscopy imaging of tracheary elements in the roots of three Arabidopsis seedlings harboring proMC9::MC9:mCherry (Bollhöner *et al.*, 2013) and proATG8a::GFP:ATG8a (Furuta *et al.*, 2014) translational fusions, after 2 h treatment with 10µM Concanamycin A to inhibit autophagosomal degradation. Correlations between GFP and mCherry signal on each pixel was calculated using the Pearson-Spearman Correlation (PCS) colocalization plugin in Image J.
(B-C) Images and quantification of GFP:ATG8a puncta (indicating autophagosomes) in these confocal laser scanning micrographs of the first differentiating xylem cells in a cell file (from the root tip) displaying detectable levels of GFP:ATG8a (driven by the pro*ATG8a* endogenous promoter active in TEs, as in (A)). Both pro*ATG8a::GFP:ATG8a* marker lines in wild-type background, or introgressed in *mc9-2* (B) or pro*IRX1::ATG5* TE autophagy overexpressor (C) were observed after propidium iodide staining (allowing to recognize TEs based on their cell wall patters, Escamez *et al.*, 2016), without or after 3h treatment with 10µM Concanamycin A (Con. A), which stops autophagic fluxes and allows accumulation of autophagosomes (*Escamez et al.*, 2016). Values that do not share any letter are significantly different ($p < 0.05$) according to a post-ANOVA Fisher test (n = 3-8 biological replicates). The displayed micrographs represent the GFP-ATG8a detection channel after thresholding of the fluorescence signal to help distinguishing autophagosomes from the diffuse GFP signal typical for GFP-ATG8 markers. The white shape outlines the part of the recorded TE that is in the analyzed focal plane (as the cells are neither flat nor perfectly straight compared with the lens, the entire cell surface is rarely entirely visible in a single focal plane). Bars = 5µm.

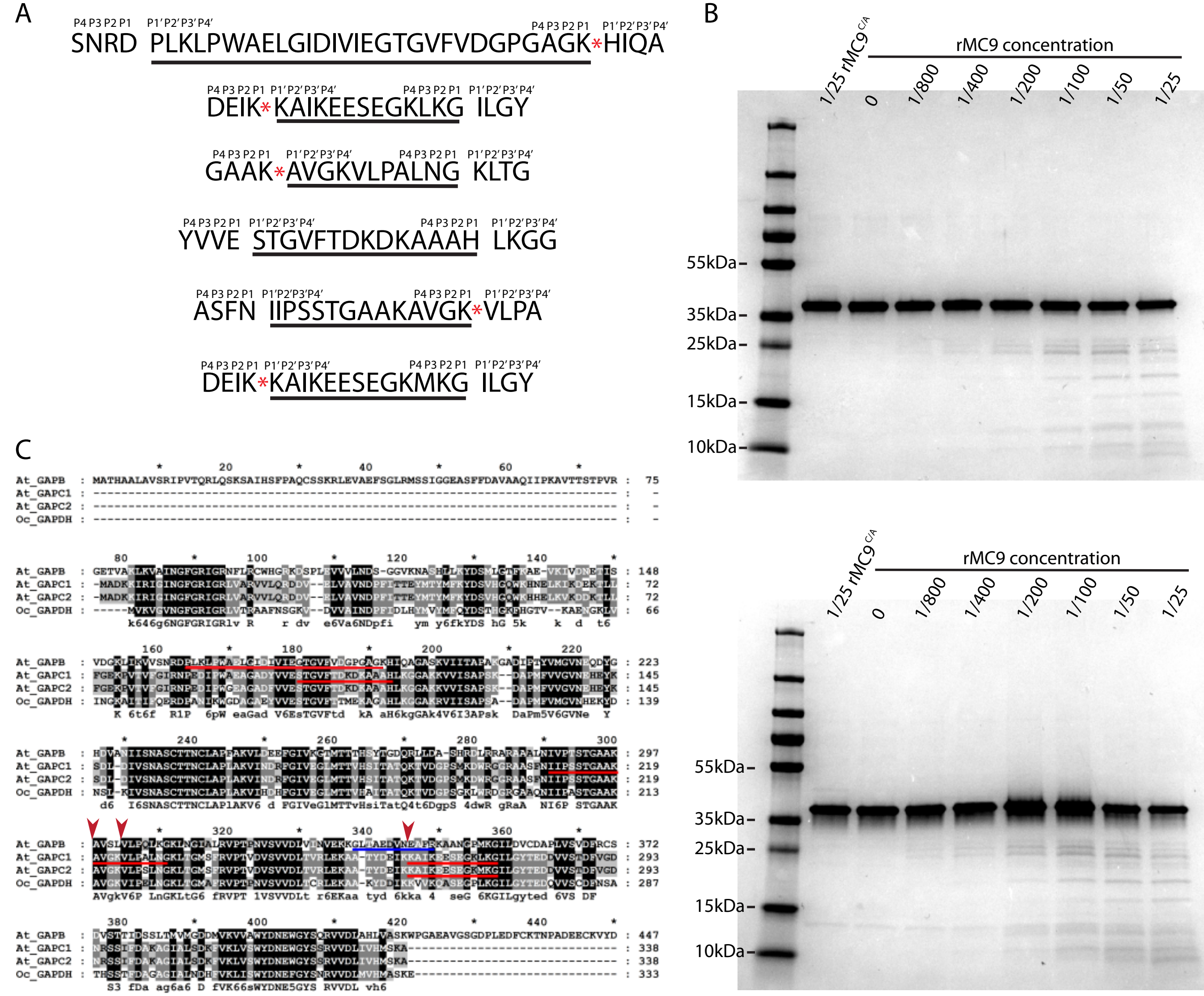

Figure S9: GAPDH peptides are detected in extracellular peptidomics of differentiating cells, possible from cleavage of GAPDH by MC9.

(A) Sequences of the six peptides originating from three GAPDH subunits (underlined) with the four amino acids from the corresponding precursor proteins around both the N-terminal and C-terminal cleavage sites (indicated by spaces). Red asterisks indicate cleavage sites compatible with METACASPASE9 favoured target sequences (Henry *et al.*, 2015).

(B) Commercially available GAPDH proteins (top GAPDH solution from rabbit muscle, product name G5262 from Sigma; bottom lyophilized GAPDH from rabbit muscle, product name G2267 from Sigma) were incubated with increasing amounts of recombinant MC9 (rMC9) or with an inactive version MC9 $^{C/A}$, as control, for 30 minutes at 30 C.

(C) Multiple sequence alignment of the *Arabidopsis thaliana* GAPDH proteins (At_GAPB, At_GAPC1 and At_GAPC2) identified in this study with Oryctolagus cuniculus (rabbit) GAPDH (Oc_GAPDH; UniProtKB - P46406). Peptides identified in this study are underlined in red. A neo-N-terminal peptide previouslt identified as potential substrate of rMC9 in (Tsiatsiani *et al.*, 2013) is underlined in blue. Red arrowheads indicate potential MC9 cleavage sites based on known MC9 recognition sites and on the observation of peptides in the peptidomics analysis in this study derived from cleavage at the indicated positions (A).

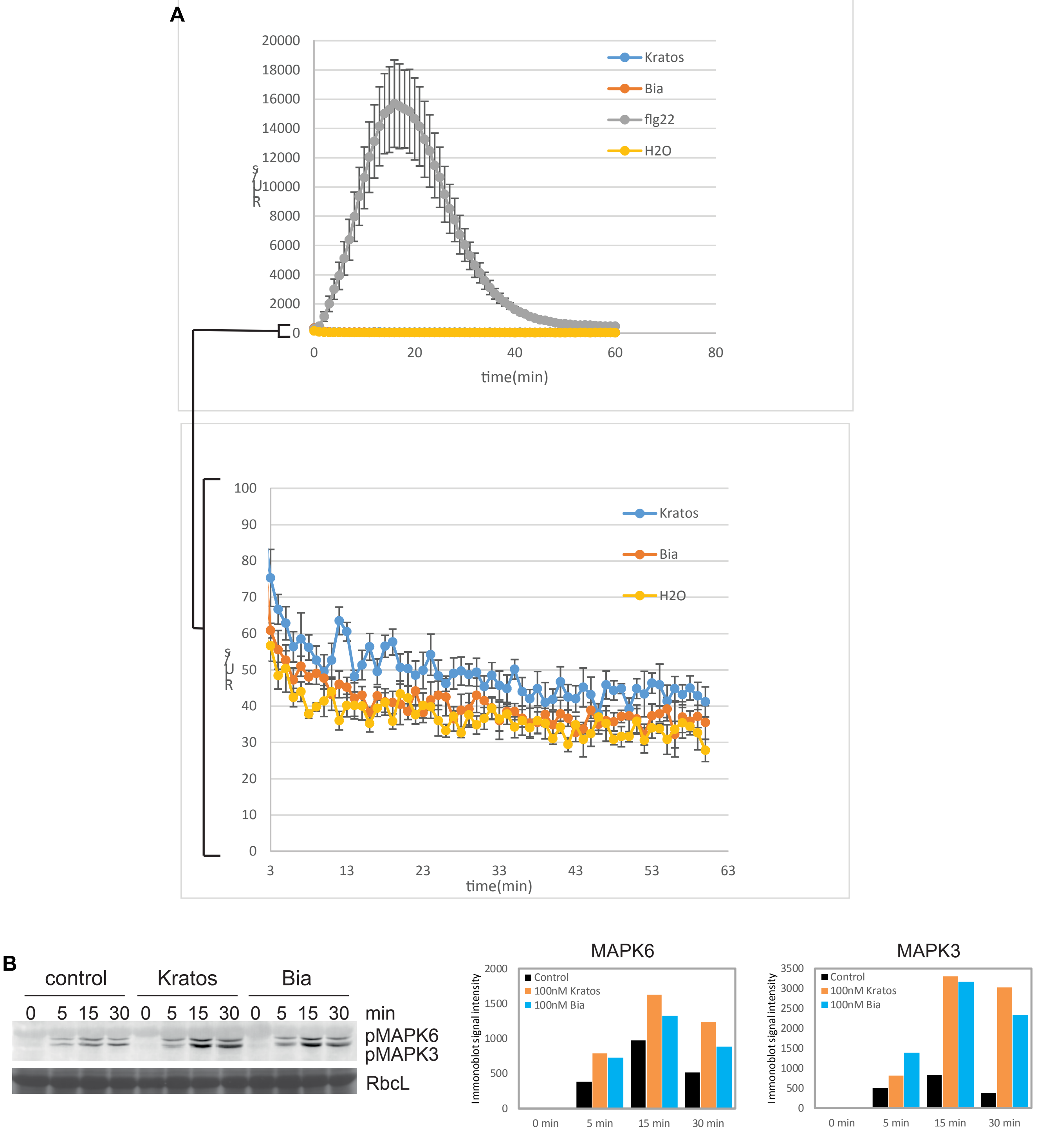

Figure S10: Kratos and Bia do not trigger oxidative burst.

(A) Reactive Oxygen Species (ROS) burst measurements in response to treatments with the peptides Kratos, Bia and Flg22 (all 100 nM). Flg22 was used as a positive control, while water was used as a negative control.

(B) Immunoblot (left) and the corresponding quantification (right) of mitogen-activated protein kinase (MAPK) activation in response to peptide infiltration (100 nM) to Col-0 rosette leaves. The control is constituted by infiltration with buffer alone (10 mM sodium phosphate, pH 7). Immunoblot of the large subunit of Rubisco (RbcL) was used as a loading control.
