# Supplementary material for: Extracellular peptide Kratos restricts cell death during vascular development and stress in Arabidopsis
Source: J Exp Bot. 2019 Feb 7;70(7):2199–210. doi: 10.1093/jxb/erz021 (PMC6460963; doi:10.1093/jxb/erz021)
Supplement: Supplementary Table S1 [file erz021_suppl_supplementary_table_s1.pdf]

Table S1. List of the unique peptides selected for analyses during vascular differentiation.

Peptide ID (in this study)	Peptide sequence	Gene ID	Gene annotation	Reason(s) for selecting this candidate	Vascular expression*
**Kratos**/Peptide 1	**GGIGKGGGIGGGIGKGGGIG**	AT3G23450	unknown function	Significant difference in peptide analysis (Dataset S1). Abundant in induced wild-type but nearly absent in *MC9* -RNAi. The full protein seems to be a repeat of this peptide.	Yes
Peptide 2	**DSTQIHTHM**	AT5G17920	Methione Synthesis	Pepetide abundant in induced *MC9*- RNAi but not found in wild-type.	Yes
Peptide 3	**AVYQRSGGAPGGAGGE**	AT5G28540	BIP1, Luminal-binding protein 1, HSP70	Pepetide abundant in induced *MC9*- RNAi but not found in wild-type.	Yes
Peptide 4	**FYVVTDPRDDNPVNPRPGT**	AT1G04680	Pectate Lyase, PLA1, lateral root emergence	Pepetide abundant in induced *MC9* -RNAi but not found in wild-type. The corresponding gene is involved in lateral root emergence, a process which also involves MC9.	Yes
Peptide 5	**LVIIPAGVPRKPG**	AT1G53240	MITOCHONDRIAL MALATE DEHYDROGENASE 1 (mMDH1)	Pepetide abundant in induced *MC9* -RNAi but not found in wild-type.	Yes
Peptide 6	**YVSIPIEGPYKPPH**	AT4G13940	HOMOLOGY-DEPENDENT GENE SILENCING 1 (HOG1)	Pepetide indentified in all conditions and genotypes except in induced *MC9*- RNAi.	Yes
Peptide 7	**TTDFPKNPPH**	AT2G38080	IRREGULAR XYLEM 12 (IRX12), laccase activity, Laccase-4	Peptide abundant only under the induced condition. The full protein is known to localize to the apoplast. The peptide contains 3 prolines, which is common for signaling peptides.	Yes
Peptide 8	**APPPMSDAHTINGKPGPL**	AT5G03260	LACCASE 11 (LAC11)	Peptide abundant only under the induced condition. The full protein is known to localize to the apoplast. The peptide contains 4 prolines, which is common for signaling peptides.	Yes
Peptide 9	**VGDVTATIVGGLNSQNPGIQK**	AT3G10080	RmlC-like cupins superfamily protein	Peptide abundant only under the controlled condition, mostly in the *MC9* -RNAi genotype.	Yes
Peptide 10	**IVNSFKTIDG**	AT1G04680	Pectin lyase-like superfamily protein	Peptide abundant only in the *MC9* -RNAi genotype.	Yes
Peptide 11	**SSAGQVDGKQ**	AT1G76160	SKU5 SIMILAR 5 (sks5)	Significant difference in peptide analysis (Dataset S1). Pepetide indentified in all conditions and genotypes except in induced *MC9* -RNAi.	Yes
Peptide 12	**RYSNSAGGVSGPIPGGPT**	AT1G76160	SKU5 SIMILAR 5 (sks5)	Significant difference in peptide analysis (Dataset S1). Abundant in induced wild-type but nearly absent in *MC9* -RNAi.	Yes
Peptide 13	**FETEPIGKGKNGKD**	AT2G05710	ACONITASE 3 (ACO3)	Significant difference in peptide analysis (Dataset S1). Abundant in induced wild-type but nearly absent in *MC9* -RNAi.	Yes
**Bia**/Peptide 14	**EKTAKKAAQAAAAASSGGGGGKGNNK**	AT3G24100	Uncharacterised protein family SERF	Pepetide abundant in induced *MC9* -RNAi but not found in wild-type.	**no data available**
Peptide 15	**AKADVEPKAAEAETKPSQV**	AT3G56240	COPPER CHAPERONE (CCH)	Peptide abundant only in the induced condition. Potential MC9 substrate	Yes
Peptide 16	**NAIGGRKGRIYVVTDPANDDPVNPRPGT**	AT3G27400	Pectin lyase-like superfamily protein	Peptide abundant in all conditions for all genotypes, therefore representing a potential control peptide.	Yes

* We considered as ”vascular expression” the detection of transcripts in tracheary elements (TEs) or in any cell neighboring TEs (i.e. pro-cambium, xylem-pole pericycle) in the Arabidopsis eFP Browser (Winter et al. 2007. *Plos One* **2**(8): e718) ”Root” dataset, consisting of transcriptomics data from different root cell types (Brady et al. 2007. *Science* 318(5851): 801-806; Cartwright et al. *2009*. *Bioinformatics* 25(19): 2581-2587).
